# Analysis of the causal structure of traits involved in sow lactation feed efficiency

**DOI:** 10.1186/s12711-022-00744-4

**Published:** 2022-07-26

**Authors:** Mónica Mora, Ingrid David, Hélène Gilbert, Guilherme J. M. Rosa, Juan Pablo Sánchez, Miriam Piles

**Affiliations:** 1 Institute of Agrifood Research and Technology (IRTA)-Animal Breeding and Genetics, Caldes de Montbui, Barcelona Spain; 2 GenPhySE, INRAE, INPT, Université de Toulouse, 31326 Castanet Tolosan, France; 3 Department of Animal and Dairy Sciences of University Madison, Madison, WI USA

## Abstract

**Background:**

Feed efficiency during lactation involves a set of phenotypic traits that form a complex system, with some traits exerting causal effects on the others. Information regarding such interrelationships can be used to predict the effect of external interventions on the system, and ultimately to optimize management practices and multi-trait selection strategies. Structural equation models can be used to infer the magnitude of the different causes of such interrelationships. The causal network necessary to fit structural equation models can be inferred using the inductive causation (IC) algorithm. By implementing these statistical tools, we inferred the causal association between the main energy sources and sinks involved in sow lactation feed efficiency for the first time, i.e., daily lactation feed intake (dLFI) in kg/day, daily sow weight balance (dSWB) in kg/day, daily litter weight gain (dLWG) in kg/day, daily back fat thickness balance (dBFTB) in mm/day, and sow metabolic body weight (SMBW) in kg^0.75^. Then, we tested several selection strategies based on selection indices, with or without dLFI records, to improve sow efficiency during lactation.

**Results:**

The IC algorithm using 95% highest posterior density (HPD_95%_) intervals resulted in a fully directed acyclic graph, in which dLFI and dLWG affected dSWB, the posterior mean of the corresponding structural coefficients (PM_λ_) being 0.12 and − 0.03, respectively. In turn, dSWB influenced dBFTB and SMBW, with PM_λ_ equal to 0.70 and − 1.22, respectively. Multiple indirect effects contributed to the variances and covariances among the analyzed traits, with the most relevant indirect effects being those involved in the association between dSWB and dBFTB and between dSWB and SMBW. Selection strategies with or without phenotypic information on dLFI, or that hold this trait constant, led to the same pattern and similar responses in dLFI, dSWB, and dLWG.

**Conclusions:**

Selection based on an index including only dBFTB and dLWG records can reduce dLFI, keep dSWB constant or increase it, and increase dLWG. However, a favorable response for all three traits is probably not achievable. Holding the amount of feed provided to the sows constant did not offer an advantage in terms of response over the other strategies.

**Supplementary Information:**

The online version contains supplementary material available at 10.1186/s12711-022-00744-4.

## Background

Feed efficiency in livestock has been widely studied to establish strategies to reduce feed costs while reducing emissions to the environment and making sustainable use of resources. In the case of reproductive females, increasing feed efficiency during lactation (LFE) has additional benefits in terms of economic costs as well as of animal wellbeing, because milk production is one of the most energy-demanding processes in the productive life of a sow [1]. When energy requirements during the lactation period are not met by the energy provided by feed because of limited feed intake, body reserves are mobilised [2] and, if this mobilization is excessive and/or repeated in successive cycles, it impairs subsequent reproductive performance [3], body condition, and health status of the female, which can result in early culling.

In pig production, most breeding programs include increasing feed efficiency during the growth/finish phase of production and litter size in the reproductive phase among the priority aims. Genetic improvement of these traits has had as correlated effects a reduction in appetite and feed intake capacity at fattening,which also extend to other stages of the animal’s life [4], as well as an increase in energy requirements during lactation, as a consequence of the high levels of prolificacy and piglet growth achieved. Some authors have suggested improving sow LFE through genetic selection but only a few studies have reported genetic parameters for measurements of this trait. Measures of LFE include (i) body energy balance [5]; (ii) ratio between the output and the input of energy efficiency of sows [6]; (iii) difference between the actual feed intake (FI) of the sow and that predicted from a phenotypic regression of FI on requirements for production and maintenance of body condition, i.e. residual feed intake (RFI) [4]; and (iv) RFI estimated from a genetic regression instead of the phenotypic regression defined before [7], which guarantees a null genetic correlation of RFI with the traits on which is the regression, and thus a null correlated response in these traits from selection on RFI [8]. These previous studies showed that LFE evaluated by these measures is heritable, thus allowing effective direct selection if the level of its genetic variability is sufficiently high. However, several other more efficient strategies of selection are possible, such as the use of a selection index based on its component traits with optimal economic weights, with or without inclusion of FI in the selection criteria (since FI records are expensive to obtain, especially with individually-housed sows), or selection performed under restricted feed intake conditions, as has been experimentally performed in growing pigs [9].

In order to define an optimal selection strategy to improve LFE by genetic selection, knowledge about the variability and covariances among the target traits is required at both the genetic and environmental levels. Estimates of such parameters are usually obtained by implementing a multiple trait animal model (MTAM). However, a MTAM only describes associations among traits, without retrieving information about causal relationships. Associations can be due to common factors that directly affect two traits, or a causal relationship between the two traits, or a combination of these two. In the causal relationship case, effects that affect one trait can have an indirect effect on the other trait through a causal link that can exist from the first to the second trait. Association among traits due to direct and indirect effects cannot be distinguished with an MTAM because it does not consider causal relationships between traits. Knowledge about the causal structure allows the prediction of the effect of external interventions for a trait on another trait (e.g., management practices such as feed restriction or cross-fostering) [10].

Structural equation models (SEM) [11] allow the representation of causal mechanisms between variables, in which the magnitude of causal relationships are described by model parameters called structural coefficients. Given two traits, X and Y, the causal relationships can be simultaneous if X affects Y and Y affects X, or recursive when only X affects Y or only Y affects X. Structural equation models have been used in many fields such as economics [12], social statistics, and biology [13]. In the last years, SEM have been increasingly implemented in quantitative genetics, following publication of the paper by Gianola and Sorensen [11], who adapted these models to this field. Regarding feed efficiency in livestock, to the best of our knowledge, only Abdalla et al. [14] inferred causal structures among feed efficiency traits in a commercial turkey population and only Wu et al. [15] implemented an SEM for FI and energy sinks in dairy cattle, assuming that energy sink traits affect FI and that there is no causal relationship between energy sinks. No studies are available on causal relationships for FE in pigs.

Components of LFE in sows have been described by Bergsma et al. [6]. The energy flows from inputs, i.e. feed intake and mobilization of energy and nutrients from body reserves, to outputs, i.e. milk yield for piglet growth and maintenance, deposition of nutrients (body weight and backfat gain), and maintenance of the sow’s physiological functions. Mobilization and deposition of energy reserves and nutrients can be summarised in a trait named ‘energy and nutrient balance’, which quantifies if the energy intakes are lower or higher than the sow’s needs during lactation. This trait could be used in a selection index with FI to prevent potential negative effects on rebreeding performance due to a negative energy balance, as suggested by Young et al. [5].

The objectives of our study were: (i) to investigate the potential causal effects that underlie the complex relationships between the main traits involved in LFE in sows, and (ii) to propose a selection strategy, with or without the inclusion of FI records, to improve sow productivity without impairing its body condition, as the latter is the main driver for future reproductive success.

## Methods

### Animals and data

The data used for this analysis cover the first 10 generations of an experiment of divergent selection for RFI in growing pigs. The detailed selection process is in Gilbert et al. [16]. Information from 1100 sows (527 and 573 from the high and low RFI lines, respectively) was recorded on two farms from 2000 to 2015. From generations 1 to 4, only third-parity batches were inseminated with Piétrain boars, and starting from generation 5 only first-parity batches were inseminated with Piétrain boars. All other batches were inseminated with RFI boars from the own line. Details on how the lactation traits were recorded are in Gilbert et al. [4].

Sow weight was recorded at entrance to maternity in the farrowing house (SW_E_) and at exit from maternity on weaning day (SW_W_), with an average duration of farrowing to weaning of 28.15 days. Backfat thickness was measured at the same time (BFT_E_ and BFT_W_, at entrance and weaning, respectively). Sow weight at farrowing (SW_F_) was estimated as in Bergsma et al. [6], using the formulas proposed by Noblet et al. [2]:

$${\mathrm{SW}}_{\mathrm{F}}\left(\mathrm{kg}\right)={\mathrm{SW}}_{\mathrm{E}}\left(\mathrm{kg}\right)-{\mathrm{LW}}_{\mathrm{S}}\left(\mathrm{kg}\right)\times \frac{{\mathrm{TFW}}_{\mathrm{E}}+{\mathrm{PW}}_{\mathrm{E}}+{\mathrm{IUFW}}_{\mathrm{E}}}{{\mathrm{TFW}}_{\mathrm{S}}},$$

where ${\mathrm{TFW}}_{\mathrm{E}}$ is the total fetal weight, ${\mathrm{PW}}_{\mathrm{E}}$ is the placenta weight and ${\mathrm{IUFW}}_{\mathrm{E}}$ is the intra-uterine fluid weight at entrance to maternity, ${\mathrm{LW}}_{\mathrm{S}}$ is the litter weight at the start of lactation, and ${\mathrm{TFW}}_{\mathrm{S}}$ is the total fetal weight at birth. ${\mathrm{TFW}}_{\mathrm{E}}$, ${\mathrm{PW}}_{\mathrm{E}}$, and ${\mathrm{IUFW}}_{\mathrm{E}}$ were calculated as follows:

$${\mathrm{TFW}}_{\mathrm{E}}\left(\mathrm{kg}\right)=\frac{{\mathrm{e}}^{\left(8.72962-\left(4.07466\times {\mathrm{e}}^{\left(-0.03318\times \left(\mathrm{dpregn}-45\right)\right)}\right)+0.000154\times \mathrm{ENgest}\times \mathrm{dpregn}+0.06774\times \mathrm{Nf}\right)}}{1000},$$

$${\mathrm{PW}}_{\mathrm{E}}\left(\mathrm{kg}\right)=\frac{{\mathrm{e}}^{\left(7.02746-0.95164\times {\mathrm{e}}^{\left(-0.06879\times \left(\mathrm{dpregn}-45\right)\right)}+0.000085\times \mathrm{ENgest}\times \mathrm{dpregn}+0.09335\times \mathrm{Nf}\right)}}{1000},$$

$${\mathrm{IUFW}}_{\mathrm{E}}\left(\mathrm{kg}\right)=\frac{{\mathrm{e}}^{\left(-0.2636+0.18805\times \mathrm{dpregn}-0.001189\times {\mathrm{dpregn}}^{2}+0.13194\times \mathrm{Nf}\right)}}{1000},$$

where $\mathrm{dpregn}$ is the number of days of pregnancy, $\mathrm{ENgest}$ is the net energy of total feed intake during gestation (MJ ME/d) and $\mathrm{Nf}$ is the number of fetuses, assumed to be equal to the total number of piglets born (TB).

Daily sow weight balance ($\mathrm{dSWB}$) and daily back fat thickness balance ($\mathrm{dBFTB}$) were computed as follows:

$$\mathrm{dSWB}\left(\frac{\mathrm{kg}}{\mathrm{day}} \right)=\frac{{\mathrm{SW}}_{\mathrm{W}}-{\mathrm{SW}}_{\mathrm{F}}}{\mathrm{DL}},$$

$$\mathrm{dBFTB}\left(\frac{\mathrm{mm}}{\mathrm{day}} \right)=\frac{{\mathrm{BFT}}_{\mathrm{W}}-{\mathrm{BFT}}_{\mathrm{E}}}{\mathrm{DL}},$$

where $\mathrm{DL}$ is the number of days in lactation.

Sow metabolic body weight ($\mathrm{SMBW}$) was computed as:

$$\mathrm{SMBW}={\left(\frac{{\mathrm{SW}}_{\mathrm{W}}+{\mathrm{SW}}_{\mathrm{F}}}{2}\right)}^{0.75}.$$

The total number of piglets born, dead or alive and the number of cross-fostered piglets (i.e., number of piglets transferred to another sow or adopted) were also recorded. Only records from litters in which cross-fostering was carried out within 2 days after farrowing were included in the analysis. All piglets were weighed at farrowing and at weaning. This information was used to compute litter weight at farrowing, following cross-fostering (${\mathrm{LW}}_{\mathrm{F}}$), and litter weight at weaning (${\mathrm{LW}}_{\mathrm{W}}$). Growth rate of the piglets during lactation was calculated to detect outliers. We considered a data point as an outlier when it was located outside the whiskers of the boxplot for the trait (i.e. outside 1.75 times the interquartile range above the upper quartile or below the lower quartile ($\mathrm{Q}1-1.75\times \mathrm{IQR}$ or $\mathrm{Q}3+1.75\times \mathrm{IQR}$). Piglets with abnormal growth rates and litters with more than one piglet with an abnormal growth rate were removed. On day 21 and until weaning, creep feeding was available, but creep feed intake was not recorded. To avoid the effect of creep feeding on litter weight gain at lactation, litter weight at day 21 (LW_21_) was used instead of litter weight at weaning. Litters with an abnormal average piglet weight at day 21 were removed. Daily litter weight gain ($\mathrm{dLWG}$) was computed as:

$$\mathrm{dLWG}\left(\frac{\mathrm{kg}}{\mathrm{day}} \right)=\frac{{\mathrm{LW}}_{21}-{\mathrm{LW}}_{\mathrm{F}}}{\mathrm{DL}},$$

where $\mathrm{DL}$ is the average number of days the piglets of a litter are alive, and daily litter weight at day 21 (${\mathrm{LW}}_{21}$) was computed by summing the body weight gain of each piglet in the litter during the period it was alive (i.e. from birth to day 21 of life or to death, if death occurred before day 21).

Only information from lactations longer than 26 days and shorter than 38 days was included in the analysis. The total feed intake of the sow (FI) was calculated as the sum of daily feed intake records available from farrowing until weaning. Feed intake data were missing for some days and only 27% of the lactations had complete records. The average number of days with missing intake records during lactation was 0.65 ± 3.03. Most missing days were 1 or 2 days after farrowing. Daily lactation feed intake (dLFI) was computed by dividing FI by the number of daily records available.

The traits dLFI, dSWB, dLWG, dBFTB, and SMBW were considered as the main components of LFE. After removing records with missing values for three or more of these traits, the dataset used for the analyses contained information on 1342 farrowings from 576 sows. The resulting numbers of records and descriptive statistics of the traits are in Table 1.

**Table 1:** Number of records, number of sows, mean standard deviation (SD), minimum (Min), and maximum (Max) of the traits investigated

Trait	Abbreviation	Records	Sows	Mean	SD	Min	Max
Daily lactation feed intake	dLFI (kg/day)	1230	540	4.87	1.08	1.03	8.39
Daily sow weight balance	dSWB (kg/day)	1307	574	− 0.49	0.69	− 2.72	1.82
Daily litter weight gain	dLWG (kg/day)	767	440	2.36	0.52	0.70	4.35
Daily back fat thickness balance	dBFTB (mm/day)	718	398	− 1.49	0.96	− 4.68	1.35
Sow metabolic body weight	SMBW (kg^0.75^)	1307	574	60.58	5.77	42.77	79.14

### Statistical analyses

In order to assess the potential causal relationships between the main components of LFE, the following two-step procedure was implemented in a Bayesian approach.

#### Step 1: Searching for recursive causal structures

With five traits involved (i.e., dLFI, dSWB, dLWG, dBFTB and SMBW), the number of possible causal structures was very large. Moreover, for some of these traits, no clear prior biological knowledge is available to establish the most likely causal structure of the system. Therefore, it was necessary to use algorithms that search for recursive causal structures (i.e., with no simultaneous relationships between traits) that are compatible with the joint distribution of the data. Because causal relationships can be masked by genetic covariances, such a search was performed on the joint distribution of the phenotypes conditional to unobservable genetic and permanent environmental effects, as proposed by Valente et al. [10]. Information on such a distribution is provided by the posterior covariance matrix of the phenotypes given the genetic and permanent environmental effects. Samples of this matrix correspond to samples of the residual covariance matrix obtained by implementing a MTAM using Gibbs sampling. The model implemented for the five traits involved in LFE was:

$${\mathbf{y}}_{{\varvec{i}}}={\mathbf{X}}_{{\varvec{i}}}\mathbf{b}+{\mathbf{a}}_{{\varvec{i}}}+{\mathbf{p}}_{{\varvec{i}}}+{\mathbf{c}\mathbf{g}}_{{\varvec{i}}}+{\mathbf{e}}_{{\varvec{i}}},$$

where ${\mathbf{y}}_{{\varvec{i}}}$ is a (5 $\times$ 1) vector of phenotypic data corresponding to individual $i$; $\mathbf{b}$ is the vector of the systematic environmental effects, including the effects of farm (with 2 levels), parity order (with 5 levels: 1, 2, 3, 4, > 4), and a 2-degree polynomial of litter size at day 21 (LS_21_) as covariates. The model for dLWG included the additional systematic effect of sire breed (with 2 levels, Piétrain or RFI lines). ${\mathbf{a}}_{{\varvec{i}}}$, ${\mathbf{p}}_{{\varvec{i}}}$, ${\mathbf{c}\mathbf{g}}_{{\varvec{i}}}$ and ${\mathbf{e}}_{{\varvec{i}}}$ are (5 $\times$ 1) vectors of additive genetic effects, permanent environmental effects associated with the sow, contemporary group effects, and residual effects, respectively, all associated with individual $i$. ${\mathbf{X}}_{{\varvec{i}}}$ $\mathrm{is}$ the incidence matrix relating individual records to systematic effects.

The joint distribution assumed for ${\mathbf{a}}_{{\varvec{i}}}$, ${\mathbf{p}}_{{\varvec{i}}}$, ${\mathbf{c}\mathbf{g}}_{{\varvec{i}}}$, and ${\mathbf{e}}_{{\varvec{i}}}$ was:

$$\left[\begin{array}{c}{\mathbf{a}}_{{\varvec{i}}}\\ {\mathbf{p}}_{{\varvec{i}}}\\ {\mathbf{c}\mathbf{g}}_{{\varvec{i}}}\\ {\mathbf{e}}_{{\varvec{i}}}\end{array}\right] \sim N \left\{\left[\begin{array}{c}\mathbf{0}\\ \mathbf{0}\\ \mathbf{0}\\ \mathbf{0}\end{array}\right],\left[\begin{array}{cccc}{\mathbf{G}}_{\mathbf{0}}& \mathbf{0}& \mathbf{0}& \mathbf{0}\\ \mathbf{0}& {\mathbf{P}}_{\mathbf{0}}& \mathbf{0}& \mathbf{0}\\ \mathbf{0}& \mathbf{0}& {\mathbf{B}}_{\mathbf{0}}& \mathbf{0}\\ \mathbf{0}& \mathbf{0}& \mathbf{0}& {\mathbf{R}}_{\mathbf{0}}\end{array}\right] \right\},$$

where ${\mathbf{G}}_{\mathbf{0}}$, ${\mathbf{P}}_{\mathbf{0}}$, ${\mathbf{B}}_{\mathbf{0}},$ and ${\mathbf{R}}_{\mathbf{0}}$ are the additive genetic, permanent environmental, contemporary group, and residual (5 $\times$ 5) covariance matrices, respectively.

The model for $n$ sows is described by:

$$\mathbf{y}=\mathbf{X}\mathbf{b}+{\mathbf{Z}}_{\mathbf{1}}\mathbf{a}+{\mathbf{Z}}_{\mathbf{2}}\mathbf{p}+{\mathbf{Z}}_{\mathbf{3}}\mathbf{c}\mathbf{g}+\mathbf{e},$$

and the joint distribution for vectors $\mathbf{a}$, $\mathbf{p}$, $\mathbf{c}\mathbf{g}$**,** and $\mathbf{e}$ is:

$$\left[\begin{array}{c}\mathbf{a}\\ \mathbf{p}\\ \mathbf{c}\mathbf{g}\\ \mathbf{e}\end{array}\right] \sim N \left\{\left[\begin{array}{c} \mathbf{0}\\ \mathbf{0}\\ \mathbf{0}\\ \mathbf{0}\end{array}\right], \left[\begin{array}{cccc}{\mathbf{G}}_{\mathbf{0}}\otimes \mathbf{A}& \mathbf{0}& \mathbf{0}& \mathbf{0}\\ \mathbf{0}& {\mathbf{P}}_{\mathbf{0}}\otimes {\mathbf{I}}_{\mathrm{n}}& \mathbf{0}& \mathbf{0}\\ \mathbf{0}& \mathbf{0}& {\mathbf{B}}_{\mathbf{0}}\otimes {\mathbf{I}}_{\mathrm{n}}& \mathbf{0}\\ \mathbf{0}& \mathbf{0}& \mathbf{0}& {\mathbf{R}}_{\mathbf{0}}\otimes {\mathbf{I}}_{\mathrm{n}}\end{array}\right]\right\},$$

where $\mathbf{y}$, $\mathbf{b}$, $\mathbf{a}$, $\mathbf{p}$, $\mathbf{c}\mathbf{g},$ and $\mathbf{e}$ are vectors of phenotypic records, systematic effects, additive genetic effects, permanent effects, contemporary group effects, and residual effects, respectively, sorted by trait and animal within trait, and $\mathbf{X}$, ${\mathbf{Z}}_{\mathbf{1}}$, ${\mathbf{Z}}_{\mathbf{2}},$ and ${\mathbf{Z}}_{\mathbf{3}}$ are known incidence matrices relating data to levels of systematic, additive genetic, permanent and contemporary group environmental effects, respectively, ${\mathbf{I}}_{\mathrm{n}}$ is the identity matrix of dimension $n$, $\otimes$ denotes the Kronecker product, and the co-variance matrices for the additive genetic effects, permanent effects, contemporary group effects, and residual effects are, respectively, $\mathbf{G}={\mathbf{G}}_{\mathbf{0}}\otimes \mathbf{A}$, ${\mathbf{P}=\mathbf{P}}_{\mathbf{0}}\otimes {\mathbf{I}}_{\mathrm{n}}$, ${\mathbf{B}=\mathbf{B}}_{\mathbf{0}}\otimes {\mathbf{I}}_{\mathrm{n}}$ and ${\mathbf{R}=\mathbf{R}}_{\mathbf{0}}\otimes {\mathbf{I}}_{\mathrm{n}}$.

The joint posterior distribution of all unknowns in the model is:

$$\mathrm{p}\left(\mathbf{b}, \mathbf{a}, \mathbf{p}, \mathbf{c}\mathbf{g}, {\mathbf{G}}_{\mathbf{0}}, {\mathbf{P}}_{\mathbf{0}}, {\mathbf{B}}_{\mathbf{0}}, {\mathbf{R}}_{\mathbf{0}}|\mathbf{y}\right)\propto \mathrm{p}\left(\mathbf{y}|\mathbf{b}, \mathbf{a}, \mathbf{p}, \mathbf{c}\mathbf{g}, {\mathbf{G}}_{\mathbf{0}}, {\mathbf{P}}_{\mathbf{0}}, {\mathbf{B}}_{\mathbf{0}}, {\mathbf{R}}_{\mathbf{0}}\right)\mathrm{p}\left(\mathbf{b}, \mathbf{a}, \mathbf{p}, \mathbf{c}\mathbf{g}, {\mathbf{G}}_{\mathbf{0}}, {\mathbf{P}}_{\mathbf{0}}, {\mathbf{B}}_{\mathbf{0}}, {\mathbf{R}}_{\mathbf{0}}\right),$$

where $\mathrm{p}\left(\mathbf{b}, \mathbf{a}, \mathbf{p}, \mathbf{c}\mathbf{g}, {\mathbf{G}}_{\mathbf{0}}, {\mathbf{P}}_{\mathbf{0}}, {\mathbf{B}}_{\mathbf{0}}, {\mathbf{R}}_{\mathbf{0}}\right)$ is equal to:

$$\mathrm{p}\left(\mathbf{b}\right)\mathrm{p}\left(\mathbf{a}|{\mathbf{G}}_{\mathbf{0}}\right)\mathrm{p}\left({\mathbf{G}}_{\mathbf{0}}\right)\mathrm{p}\left(\mathbf{p}|{\mathbf{P}}_{\mathbf{0}}\right)\mathrm{p}\left({\mathbf{P}}_{\mathbf{0}}\right)\left(\mathbf{c}\mathbf{g}|{\mathbf{B}}_{\mathbf{0}}\right)\mathrm{p}\left({\mathbf{B}}_{\mathbf{0}}\right)\left(\mathbf{e}|{\mathbf{R}}_{\mathbf{0}}\right)\mathrm{p}\left({\mathbf{R}}_{\mathbf{0}}\right).$$

We assumed standard prior distributions for all the unknowns, as reported in Sorensen and Gianola [17].

The inductive causation (IC) algorithm was applied to samples of the residual covariance matrix using a program written in R [18] by Valente and Rosa [19]. This algorithm performs a series of statistical decisions based on partial correlations between pairs of traits in a three-step procedure, as described in Valente et al. [10]. For each partial correlation, the highest posterior density (HPD) interval with some specified probability content was computed. If the HPD interval contains 0, the correlation was declared null. Otherwise, the two variables involved were declared as conditionally dependent. In this study, we applied HPD content magnitudes of 70, 80, 90, and 95%, and compared the causal structures obtained with each of them. The output of the IC algorithm is typically a directed acyclic graph (DAG), which consists of a set of variables (symbolized by nodes) connected by directed edges (symbolized by arrows), which represent direct causal relationships.

#### Step 2: Fitting a SEM

Once the recursive causal structure was established, a SEM with that specific structure was fitted. The SEM for the $n$ sows is described by:

$$\mathbf{y}=\left({\varvec{\Lambda}}\otimes {\mathbf{I}}_{\mathrm{n}}\right)\mathbf{y}+\mathbf{X}{\mathbf{b}}_{\mathbf{S}}+{\mathbf{Z}}_{\mathbf{1}}{\mathbf{a}}_{\mathbf{S}}+{\mathbf{Z}}_{\mathbf{2}}{\mathbf{p}}_{\mathbf{S}}+{\mathbf{Z}}_{\mathbf{3}}{\mathbf{c}\mathbf{g}}_{\mathbf{S}}+{\mathbf{e}}_{\mathbf{S}},$$

where ${\varvec{\Lambda}}$ is a (5 $\times$ 5) matrix with 0 s on the diagonal and with structural coefficients ($\lambda$) for the off-diagonal elements. Each non-null entry of the matrix expresses the magnitude of the causal effect of the trait corresponding to its column on the trait corresponding to its row. The other terms of the SEM were the same as those described for the above MTAM. However, to distinguish the terms of the MTAM from those of the SEM, the latter will be denoted with the sub-index $s$. The residual (co)variance matrix in SEM was constructed as diagonal to achieve identifiability of all model parameters for any acyclic structure, following Wu et al.[20].

As shown by Gianola and Sorensen [11], the model in the previous equation can be rewritten as:

$$\mathbf{y}={\left[{\mathbf{I}}_{5\mathrm{n}}-\left({\varvec{\Lambda}}\otimes {\mathbf{I}}_{\mathrm{n}}\right)\right]}^{-1}\mathbf{X}{\mathbf{b}}_{\mathbf{S}}+{\left[{\mathbf{I}}_{5\mathrm{n}}-\left({\varvec{\Lambda}}\otimes {\mathbf{I}}_{\mathrm{n}}\right)\right]}^{-1}{\mathbf{Z}}_{\mathbf{1}}{\mathbf{a}}_{\mathbf{S}} +{\left[{\mathbf{I}}_{5\mathrm{n}}-\left({\varvec{\Lambda}}\otimes  {\mathbf{I}}_{\mathrm{n}}\right)\right]}^{-1}{\mathbf{Z}}_{\mathbf{2}}{\mathbf{p}}_{\mathbf{S}}+{\left[{\mathbf{I}}_{5\mathrm{n}}-\left({\varvec{\Lambda}}\otimes {\mathbf{I}}_{\mathrm{n}}\right)\right]}^{-1}{\mathbf{Z}}_{\mathbf{3}}{\mathbf{c}\mathbf{g}}_{\mathbf{S}}+{\left[{\mathbf{I}}_{5\mathrm{n}}-\left({\varvec{\Lambda}}\otimes {\mathbf{I}}_{\mathrm{n}}\right)\right]}^{-1}{\mathbf{e}}_{\mathbf{S}},$$

and is named the “reduced model”, where ${\mathbf{I}}_{5\mathrm{n}}$ is a (5$n\times 5n$) identity matrix. The reduced model is equivalent to a MTAM, considering that the vectors ${\mathbf{b}}^{*}={\left[{\mathbf{I}}_{5\mathrm{n}}-\left({\varvec{\Lambda}}\otimes {\mathbf{I}}_{\mathrm{n}}\right)\right]}^{-1}{\mathbf{b}}_{\mathbf{S}}$, ${\mathbf{a}}^{*}={\left[{\mathbf{I}}_{5\mathrm{n}}-\left({\varvec{\Lambda}}\otimes {\mathbf{I}}_{\mathrm{n}}\right)\right]}^{-1}{\mathbf{a}}_{\mathbf{S}}$, ${\mathbf{p}}^{*}={\left[{\mathbf{I}}_{5\mathrm{n}}-\left({\varvec{\Lambda}}\otimes {\mathbf{I}}_{\mathrm{n}}\right)\right]}^{-1}{\mathbf{p}}_{\mathbf{S}}$, ${\mathbf{c}\mathbf{g}}^{*}={\left[{\mathbf{I}}_{5\mathrm{n}}-\left({\varvec{\Lambda}}\otimes {\mathbf{I}}_{\mathrm{n}}\right)\right]}^{-1}{\mathbf{c}\mathbf{g}}_{\mathbf{S}}$, and ${\mathbf{e}}^{*}={\left[{\mathbf{I}}_{5\mathrm{n}}-\left({\varvec{\Lambda}}\otimes {\mathbf{I}}_{\mathrm{n}}\right)\right]}^{-1}{\mathbf{e}}_{\mathbf{S}}$ correspond to the total (i.e., direct effects plus indirect effects mediated by other traits that have causal effects on the traits) systematic, genetic, permanent environmental, contemporary group, and residual effects obtained from the MTAM. Therefore, the covariance matrices ${\mathbf{G}}_{\mathbf{0}}^{\mathbf{*}}={\left[{\mathbf{I}}_{5}-{\varvec{\Lambda}}\right]}^{-1}{\mathbf{G}}_{\mathbf{0},\mathbf{S}}{\left[{\mathbf{I}}_{5}-{\varvec{\Lambda}}\right]}^{\mathrm{^{\prime}}-1}$, ${\mathbf{P}}_{\mathbf{0}}^{\mathbf{*}}={\left[{\mathbf{I}}_{5}-{\varvec{\Lambda}}\right]}^{-1}{\mathbf{P}}_{\mathbf{0},\mathbf{S}}{\left[{\mathbf{I}}_{5}-{\varvec{\Lambda}}\right]}^{\mathrm{^{\prime}}-1}$, ${\mathbf{B}}_{\mathbf{0}}^{\mathbf{*}}={\left[{\mathbf{I}}_{5}-{\varvec{\Lambda}}\right]}^{-1}{\mathbf{B}}_{\mathbf{0},\mathbf{S}}{\left[{\mathbf{I}}_{5}-{\varvec{\Lambda}}\right]}^{\mathrm{^{\prime}}-1}$, and ${\mathbf{R}}_{\mathbf{0}}^{\mathbf{*}}={\left[{\mathbf{I}}_{5}-{\varvec{\Lambda}}\right]}^{-1}{\mathbf{R}}_{\mathbf{0},\mathbf{S}}{\left[{\mathbf{I}}_{5}-{\varvec{\Lambda}}\right]}^{\mathrm{^{\prime}}-1}$ are those of the total additive genetic effects (${\mathbf{G}}_{\mathbf{0}}^{\mathbf{*}}$), total permanent environmental effects (${\mathbf{P}}_{\mathbf{0}}^{\mathbf{*}}$), total contemporary group effects (${\mathbf{B}}_{\mathbf{0}}^{\mathbf{*}}$), and total residuals (${\mathbf{R}}_{\mathbf{0}}^{\mathbf{*}}$). Prior distributions of SEM were the same as those for the MTAM but $\mathrm{p}\left({\mathbf{R}}_{\mathbf{0},\mathrm{S}}\right)\sim \prod_{j=1}^{5}P({\sigma }_{j}^{2})$ and $\mathrm{p}\left({\varvec{\Lambda}}\right)\sim k (\mathrm{constant})$.

All analyses were performed with the Gibbs sampling algorithm using the Gibbsf90 software [21]. Single sampling processes of 2,000,000 iterations were run for all models, discarding the first 300,000 iterations based on visual inspection of trace plots of each chain and saving 1 of every 100 samples. Checking for convergence of the sampling process after 300,000 iterations according to Geweke’s criterion [22] was performed using the”boa” R package [23]. Posterior marginal inferences of variance components were performed with the Postgibbsf90 software [21].

### Selection strategies

All proposed genetic selection strategies were based on selection indices including only sow’s phenotypes with or without constraints and for which the objective was always to increase dLWG, either by reducing or keeping dLFI constant, and either by increasing or keeping dSWB constant. Since there is no published information on economic weights of the analyzed traits, the selection index coefficients were computed for different combinations of three arbitrary values (0, 1, 2) for economic values, with the appropriate sign according to the selection objective. Different selection strategies were defined based on the traits that were recorded on the nucleus farm (Table 2). For dLFI, strategies with or without recording of this trait and inclusion in the selection criterion were considered. An additional selection strategy that consisted in restricted feeding (i.e., the amount of food provided to the sow was kept constant over the lactation) for all sows was also considered. In this case, the genetic and phenotypic variances of dLFI were set equal to zero in the genetic and phenotypic matrices of the “reduced model” in order to remove the contribution of dLFI to FE, such that the variation in feed efficiency depended only on variation in dSWB, dBFTB, and dLWG, which are easier to measure than dLFI. Keeping the amount of feed provided to all the sows constant has an impact on the causal structure because then indirect effects of dLFI no longer contribute to variation in the other traits. Knowledge of causal relationships between the traits allows predicting the effect on selection response when this external intervention is applied, without the need to perform a new experiment and data analysis to estimate variance components under those environmental conditions.

**Table 2:** Scheme of the different selection strategies

Strategy	dLFI	dSWB	dBFTB	dLWG
Strategy 1	Recorded	Recorded	Not recorded	Recorded
Strategy 2	Not recorded	Recorded	Not recorded	Recorded
Strategy 3	Restricted	Recorded	Not Recorded	Recorded
Strategy 4	Recorded	Not recorded	Recorded	Recorded
Strategy 5	Not recorded	Not recorded	Recorded	Recorded
Strategy 6	Restricted	Not recorded	Recorded	Recorded

Let $\mathbf{y}\mathbf{^{\prime}}=\left\{{\mathrm{y}}_{1}, {\mathrm{y}}_{2}, \dots , {\mathrm{y}}_{\mathrm{m}}\right\}$ denote the vector of the set of $\mathrm{m}$ traits to be improved, with economic values $\mathbf{a}\mathbf{^{\prime}}=\left\{{\mathrm{a}}_{1}, {\mathrm{a}}_{2},\dots , {\mathrm{a}}_{\mathrm{m}}\right\}$. Then, the selection objective is defined as $\mathrm{H}=\mathbf{a}\mathbf{^{\prime}}\mathbf{y}$. Let $\mathbf{x}\mathbf{^{\prime}}=\left\{{\mathrm{x}}_{1}, {\mathrm{x}}_{2}, \dots , {\mathrm{x}}_{\mathrm{k}}\right\}$ be the vector of records corresponding to the set of k traits measured on each sow to predict breeding values. The selection index is $\mathrm{I}=\mathbf{b}\mathbf{^{\prime}}\mathbf{x}$, with coefficients computed as $\mathbf{b}={\mathbf{P}}^{-1}\mathbf{G}\mathbf{a}$. In this expression, $\mathbf{P}$ is the total (i.e. obtained from the “reduced model”) phenotypic covariance matrix between the traits included in the selection criterion of dimension ($\mathrm{k }\times \mathrm{ k}$), $\mathbf{G}$ is the total genetic covariance matrix between the traits in the selection criterion and the traits in the selection of dimension ($\mathrm{k }\times \mathrm{ m}$), and $\mathbf{b}$ and $\mathbf{a}$ are as defined before. Response to selection based on the selection criterion $\mathrm{I}$ for a trait ${\mathrm{y}}_{\mathrm{j}}$ in the selection objective is ${\mathrm{CR}}_{\mathrm{j}}={\mathrm{i}}_{\mathrm{I}}\frac{{\mathbf{b}}^{\mathbf{^{\prime}}}{\mathbf{G}}_{\mathbf{j}}}{\sqrt{{\mathbf{b}}^{\mathbf{^{\prime}}}\mathbf{P}\mathbf{b}}}$, where ${\mathbf{G}}_{\mathbf{j}}$ is the $\mathrm{j}$th column of matrix $\mathbf{G}$ that includes the genetic covariances of the traits in $\mathrm{I}$ with trait ${\mathrm{y}}_{\mathrm{j}}$ and ${\mathrm{i}}_{\mathrm{I}}$ is the selection intensity on the index.

When the correlated response of a particular trait (${\mathrm{y}}_{\mathrm{r}}$) is constrained to zero, i.e. the selection criterion results in zero genetic change for a particular trait, the coefficients of the selection index were computed following Brascamp [24]:

$$\mathbf{b}={\mathbf{P}}^{-\mathbf{1}}\left[\mathbf{I}-{\mathbf{G}}_{\mathbf{r}}{\left({\mathbf{G}}_{\mathbf{r}}^{\mathrm{^{\prime}}}{\mathbf{P}}^{-\mathbf{1}}{\mathbf{G}}_{\mathbf{r}}\right)}^{-\mathbf{1}}{\mathbf{G}}_{\mathbf{r}}^{\mathrm{^{\prime}}}{\mathbf{P}}^{-\mathbf{1}}\right]\mathbf{G}\mathbf{a},$$

where ${\mathbf{G}}_{\mathbf{r}}$ is the $\mathrm{r}$th column of the matrix $\mathbf{G}$ that includes the genetic covariances of the traits in $\mathrm{I}$ with trait ${\mathrm{y}}_{\mathrm{r}}$ and all other terms are as defined before.

In all selection indices, economic weights were assumed to be equal to − 2, − 1 or 0 for dLFI, + 2, + 1 or 0 for dSWB, and + 2 or + 1 for dLWG. The intensity of selection was set equal to 1.3.

## Results

### Recursive causal structure

**Fig. 1:**
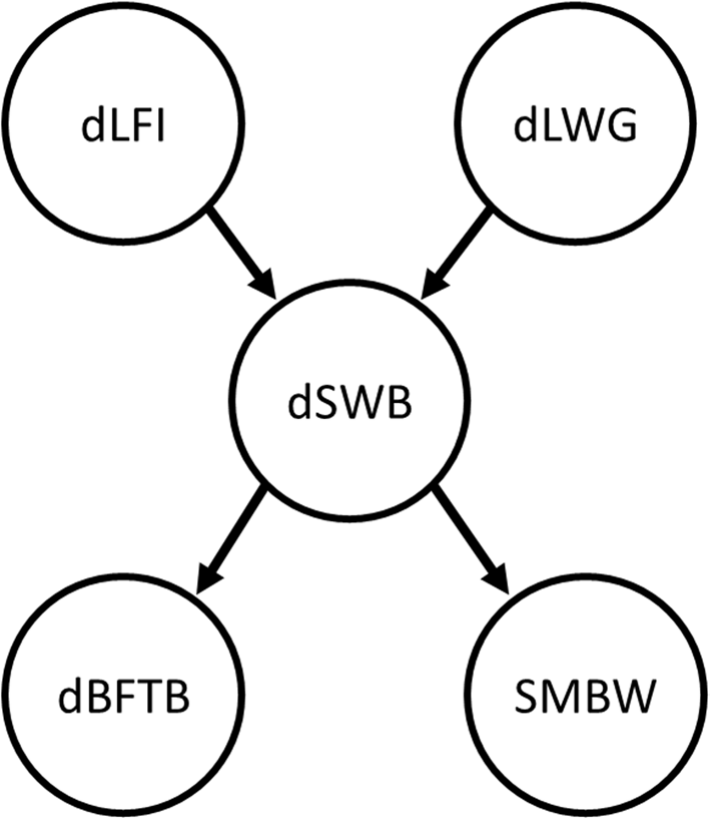
Causal structure between daily lactation feed intake (dLFI), daily sow weight balance (dSWB), daily litter weight gain (dLWG), daily back fat thickness balance (dBFTB) and sow metabolic body weight (SMBW) based on the inductive causation algorithm with statistical decisions made using highest posterior density intervals with 95% probability content. ${\uplambda }_{\mathrm{i}\leftarrow \mathrm{j}}$ denotes a structural coefficient and represents the effect of trait $\mathrm{j}$ on trait $\mathrm{i}$

High posterior density intervals of 70, 80, 90, and 95% probability were used for decisions in the IC algorithm to assess causal relationships among the five analyzed traits. In the first three cases, the same edges were obtained but no direction was established. However, when 95% HPD intervals (HPD_95%_) were used, a DAG was returned with the same edges as obtained for the other HPD, but in this case, the edge between dLFI and dLWG disappeared (Fig. 1). This last causal structure indicated that dLFI and dLWG affected dSWB, measured by structural coefficients denoted by ${\lambda }_{dSWB\leftarrow dLFI}$ and ${\lambda }_{dSWB\leftarrow dLWG}$, respectively, and that, in turn, dSWB had an effect on dBFTB and on SMBW, denoted by ${\lambda }_{dBFTB\leftarrow dSWB}$ and ${\lambda }_{SMBW\leftarrow dSWB}$, respectively.

The causal structure returned by the IC algorithm based on HPD_95%_ intervals was implemented in the SEM because all directions were found in this case. Posterior means and HPD_95%_ of the structural coefficients are in Table 3. Since HPD_95%_ regions did not include 0, all structural coefficients were statistically different from 0. Daily lactation feed intake had a positive effect on dSWB. Thus, an increase of 1 kg/day in dLFI on average produces an increase of 0.12 kg/day on dSWB. However, dLWG had an unfavorable but small effect on dSWB, decreasing its value by 0.03 kg/day for every 1 kg/day increase in dLWG. Daily sow weight balance, in turn, had a favorable effect on both dBFTB and SMBW, increasing the value of dBFTB by 0.07 mm/day and decreasing SMBW by 1.22 kg for each kg/day increase in dSWB.

**Table 3:** Posterior means and 95% highest posterior density (HPD_95%_) intervals of structural coefficients pertaining to the structural equation model that results from implementation of the inductive causation algorithm based on HPD_95%_ intervals

Structural coefficient^a^	Posterior mean [HPD_95%_]	Standardized posterior mean^b^
${\lambda }_{dSWB\leftarrow dLFI}$	0.12 [0.08,0.15]	0.30 [0.22,0.39]
${\lambda }_{dSWB\leftarrow dLWG}$	− 0.03 [− 0.05,− 0.004]	− 0.05 [− 0.09,− 0.008]
${\lambda }_{dBFTB\leftarrow dSWB}$	0.70 [0.55,0.87]	0.47 [0.37,0.58]
${\lambda }_{SMBW\leftarrow dSWB}$	− 1.22 [− 1.59,− 0.87]	− 0.07 [− 0.09,− 0.05]

^a^${\lambda }_{i\leftarrow j}$ denotes a structural coefficient which represents the effect of trait j on trait i. dLFI = daily lactation feed intake (kg/day); dSWB (daily sow weight balance (kg/day); dLWG = daily litter weight gain (kg/day); dBFT = daily back fat thickness (mm/day); SMBW = sow metabolic body weight

^b^Standardized structural coefficient was calculated as ${\uplambda }_{\mathrm{i}\leftarrow \mathrm{j}}\frac{\mathrm{sd}(\mathrm{j})}{\mathrm{sd}(\mathrm{i})}$

### Quality of fit

The deviance information criterion (DIC) [25] was used to check the fit of the models. The value of this parameter was 2508.77 for the MTAM and 2422.83 for the SEM, which clearly suggested that the SEM was more appropriate for these data. Posterior distributions of all variance components for all traits from the “reduced model” were quite similar to the posterior distributions obtained from the MTAM, and not statistically different from each other for any component (results not shown).

### Variance components and heritabilities

Means and standard deviations (SD) of posterior marginal distributions of variance components obtained from the MTAM and SEM are in Tables 4 and 5, respectively. In the SEM, variance components reflect the direct effects of the genetic and environmental factors on a trait, while in the MTAM, they correspond to the total effects, i.e., the direct plus indirect effects mediated by other traits that have causal effects. The results from both models indicate that the analyzed traits show moderate to high heritabilities, with posterior means (posterior SD) ranging from 0.16 (0.03) for dSWB to 0.41 (0.05) for SMBW in the MTAM (total heritability), and from 0.12 (0.03) for dBFTB to 0.36 (0.06) for SMBW in the SEM (direct heritability). Estimates of variance components for dLFI and dLWG should not be statistically different between the MTAM and SEM models because they are not phenotypically affected by any other trait. However, unexpectedly the posterior means of the variance of permanent environmental effects of the sow differed between the two models. Nonetheless, the direct and total heritabilities were not statistically different between the two models for these two traits.

**Table 4:** Posterior means (posterior SD) of variance components for the traits involved in lactation feed efficiency based on the multiple trait animal model

Parameter	dLFI	dSWB	dLWG	dBFTB	SMBW
${\upsigma }_{\mathrm{a}}^{2}$	0.16 (0.02)	0.06 (0.01)	0.04 (0.008)	0.16 (0.03)	8.18 (1.49)
${\upsigma }_{\mathrm{cg}}^{2}$	0.28 (0.03)	0.10 (0.01)	0.03 (0.006)	0.21 (0.05)	3.70 (0.58)
${\upsigma }_{\mathrm{p}}^{2}$	0.03 (0.01)	0.05 (0.01)	0.02 (0.006)	0.08 (0.03)	4.99 (0.90)
${\upsigma }_{\mathrm{e}}^{2}$	0.26 (0.01)	0.17 (0.009)	0.05 (0.004)	0.41 (0.03)	3.74 (0.23)
${\mathrm{h}}^{2}$	0.21 (0.03)	0.16 (0.03)	0.27 (0.05)	0.18 (0.04)	0.41 (0.05)

dLFI = daily lactation feed intake (kg/day); dSWB = daily sow weight balance (kg/day); dLWG = daily litter weight gain (kg/day); dBFTB = daily back fat thickness balance (mm/10); SMBW = sow metabolic body weight (kg^0.75^)

${\upsigma }_{\mathrm{a}}^{2}$ = additive variance; ${\upsigma }_{\mathrm{cg}}^{2}$ = contemporary group variance; ${\upsigma }_{\mathrm{p}}^{2}$ = permanent variance; ${\upsigma }_{\mathrm{e}}^{2}$ = residual variance; ${\mathrm{h}}^{2}$ = heritability

**Table 5:** Posterior means (posterior SD) of variance components for the traits involved in lactation feed efficiency based on the structural equation model

Parameter	dLFI	dSWB	dLWG	dBFTB	SMBW
${\upsigma }_{\mathrm{a}}^{2}$	0.14 (0.03)	0.07 (0.02)	0.05 (0.009)	0.07 (0.02)	7.12 (1.40)
${\upsigma }_{\mathrm{cg}}^{2}$	0.28 (0.04)	0.12 (0.02)	0.03 (0.006)	0.17 (0.04)	3.33 (0.53)
${\upsigma }_{\mathrm{p}}^{2}$	0.07 (0.02)	0.04 (0.01)	0.008 (0.005)	0.08 (0.03)	6.01 (0.97)
${\upsigma }_{\mathrm{e}}^{2}$	0.25 (0.01)	0.14 (0.008)	0.05 (0.004)	0.29 (0.02)	3.46 (0.21)
${\mathrm{h}}^{2}$	0.19 (0.04)	0.20 (0.04)	0.35 (0.05)	0.12 (0.03)	0.36 (0.06)

dLFI = daily lactation feed intake (kg/day); dSWB (daily sow weight balance (kg/day); dLWG = daily litter weight gain (kg/day); dBFTB = daily back fat thickness balance (mm/day); SMBW = sow metabolic body weight (kg^0.75^)

${\upsigma }_{\mathrm{a}}^{2}$ = additive variance; ${\upsigma }_{\mathrm{cg}}^{2}$ = contemporary group variance; ${\upsigma }_{\mathrm{p}}^{2}$ = permanent variance; ${\upsigma }_{\mathrm{e}}^{2}$ = residual variance; ${\mathrm{h}}^{2}$ = heritability

For dSWB and dBFTB, the posterior means of the residual variances were statistically lower (HPD_95%_ intervals do not overlap) in the SEM compared to the MTAM. Likewise, the posterior mean of the additive genetic variance for dBFTB was statistically lower in the SEM than in the MTAM. All these results suggest a relevant contribution of indirect effects to the variances of these traits.

### Associations between traits

Posterior means (HPD_95%_) of residual correlations from the MTAM are in Table 6. The signs of these correlations coincide with the signs of the structural coefficients. Residual correlations between traits connected by a causal relationship were all statistically different from 0, i.e. the HPD_95%_ intervals did not include 0. All others correlations were not statistically different from 0, except for the correlation between dLFI and dBFTB.

**Table 6:** Posterior means of residual correlations and 95% highest posterior density (HPD_95%_) intervals among the traits involved in lactation feed efficiency based on the multiple trait animal model (MTAM)

Trait	Trait	Residual correlation	
		Posterior mean	HPD_95%_
dLFI	dSWB	0.48	[0.42, 0.54]
	dLWG	0.11	[− 0.009, 0.22]
	dBFTB	0.25	[0.15, 0.34]
	SMBW	− 0.21	[− 0.29, 0.13]
dSWB	dLWG	− 0.24	[− 0.35, − 0.13]
	dBFTB	0.52	[0.43, 0.60]
	SMBW	− 0.28	[− 0.35, − 0.20]
dLWG	dBFTB	− 0.13	[− 0.27, 0.01]
	SMBW	0.04	[− 0.08, 0.16]
dBFTB	SMBW	− 0.14	[− 0.40, 0.14]

dLFI = daily lactation feed intake (kg/day); dSWB (daily sow weight balance (kg/day); dLWG = daily litter weight gain (kg/day); dBFTB = daily back fat thickness balance (mm/day); SMBW = sow metabolic body weight (kg^0.75^)

Posterior means (HPD_95%_) of the genetic correlations obtained from the MTAM and SEM models are in Table 7. Estimates of genetic correlations for traits that were not directly connected in the DAG were similar for the two models, e.g. the correlations between dLFI and dLWG (0.46 [0.23,0.67] and 0.41 [0.18,0.65] for MTAM and SEM, respectively). Conversely, large differences were found for the other traits, e.g. the correlations between dLFI and dBFTB (0.46 [0.20,0.70] and 0.25 [− 0.13,0.61] for the MTAM and SEM, respectively), dLFI and SMBW (0.39 [0.14,0.64] and 0.21 [− 0.09,0.53]), and for the correlation between dSWB and dBFTB (0.69 [0.46,0.89] and 0.31 [− 0.09,0.72]). However, since the HPD_95%_ were very large, these estimates were not statistically different from each other.

**Table 7:** Posterior means of genetic correlations and 95% highest posterior density (HPD_95%_) intervals among traits based on the multiple trait animal model (MTAM) and on the structural equation model (SEM)

Trait	Trait	MTAM		SEM	
		Posterior mean	HPD_95%_	Posterior mean	HPD_95%_
dLFI	dSWB	0.51	[0.23, 0.78]	0.42	[0.15, 0.68]
	dLWG	0.46	[0.23, 0.67]	0.41	[0.18, 0.65]
	dBFTB	0.46	[0.20, 0.70]	0.25	[− 0.13, 0.61]
	SMBW	0.39	[0.14, 0.64]	0.21	[− 0.09, 0.53]
dSWB	dLWG	− 0.37	[− 0.70, − 0.06]	− 0.55	[− 0.76, − 0.31]
	dBFTB	0.69	[0.46, 0.89]	0.31	[− 0.09, 0.72]
	SMBW	− 0.28	[− 0.58, 0.02]	− 0.34	[− 0.65, − 0.04]
dLWG	dBFTB	− 0.46	[− 0.71, − 0.14]	− 0.46	[− 0.74, − 0.15]
	SMBW	0.41	[0.14, 0.66]	0.44	[0.18, 0.68]
dBFTB	SMBW	− 0.14	[− 0.40, 0.14]	− 0.06	[− 0.42, 0.31]

MTAM = multiple trait animal model; SEM = structural equation model

dLFI = daily lactation feed intake (kg/day); dSWB (daily sow weight balance (kg/day); dLWG = daily litter weight gain (kg/day); dBFTB = daily back fat thickness balance (mm/day); SMBW = sow metabolic body weight (kg^0.75^)

Estimates of correlations for permanent environmental and contemporary group effects between traits are in Additional file 1: Tables S1 and S2. As with the genetic correlations, posterior means of correlations for permanent environmental and contemporary group effects did not differ much between the two models for traits that were not connected in the DAG, but differences were larger for the other traits. However, HPD_95%_ intervals were relatively large in all cases, precluding any claim of statistical difference.

**Fig. 2:**
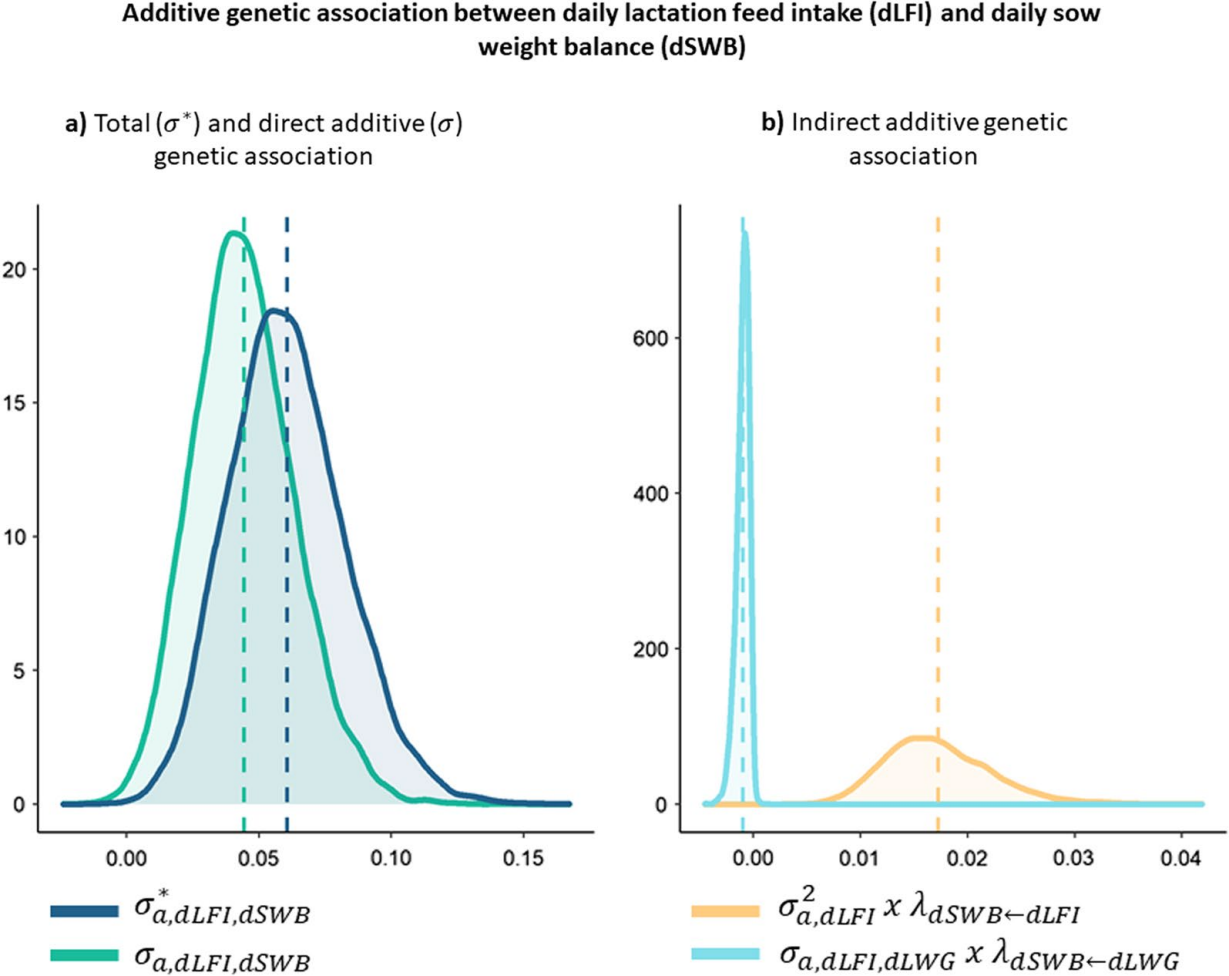
Additive genetic association between daily lactation feed intake (dLFI) and daily sow weight balance (dSWB). **a** Total (${\upsigma }^{*}$) and direct additive ($\upsigma$) genetic association and **b** indirect additive genetic association

Estimated marginal posterior distributions of total (${\upsigma }_{\mathrm{a},\mathrm{x},\mathrm{y}}^{*})$, direct (${\upsigma }_{\mathrm{a},\mathrm{x},\mathrm{y}}$), and indirect genetic associations between traits that were related by a causal association are described in the following. In Fig. 2, additive genetic associations between dLFI and dSWB are represented. The total additive genetic association in the “reduced model” between dLFI and dSWB was calculated as follows:

$${\upsigma }_{\mathrm{a},\mathrm{dLFI},\mathrm{dSWB}}^{*}= {\upsigma }_{\mathrm{a},\mathrm{dLFI}}^{2}\times {\uplambda }_{\mathrm{dSWB}\leftarrow \mathrm{dLFI}}{+\upsigma }_{\mathrm{a},\mathrm{dLFI},\mathrm{dSWB}}+{\upsigma }_{\mathrm{a},\mathrm{dLFI},\mathrm{dLWG}}\times {\uplambda }_{\mathrm{dSWB}\leftarrow \mathrm{dLWG}},$$

where ${\upsigma }_{\mathrm{a},\mathrm{dLFI},\mathrm{dSWB}}$ represents the association between the additive genetic effects that directly affect both dLFI and dSWB (pleiotropic effects), while ${\upsigma }_{\mathrm{a},\mathrm{dLFI}}^{2}\times {\uplambda }_{\mathrm{dSWB}\leftarrow \mathrm{dLFI}}$ and ${\upsigma }_{\mathrm{a},\mathrm{dLFI},\mathrm{dLWG}}\times {\uplambda }_{\mathrm{dSWB}\leftarrow \mathrm{dLWG}}$ represent the genetic associations due to indirect effects of dLFI on dSWB and of dLWG on dSWB. Posterior distributions of the total additive genetic covariance (${\upsigma }_{\mathrm{a},\mathrm{dLFI},\mathrm{dSWB}}^{*}$) and the direct additive genetic covariance (${\upsigma }_{\mathrm{a},\mathrm{dLFI},\mathrm{dSWB}}$) differed slightly from each other because of the small contribution of the terms ${\upsigma }_{\mathrm{a},\mathrm{dLFI}}^{2}\times {\uplambda }_{\mathrm{dSWB}\leftarrow \mathrm{dLFI}}$ and ${\upsigma }_{\mathrm{a},\mathrm{dLFI},\mathrm{dLWG}}\times {\uplambda }_{\mathrm{dSWB}\leftarrow \mathrm{dLWG}}$.

**Fig. 3:**
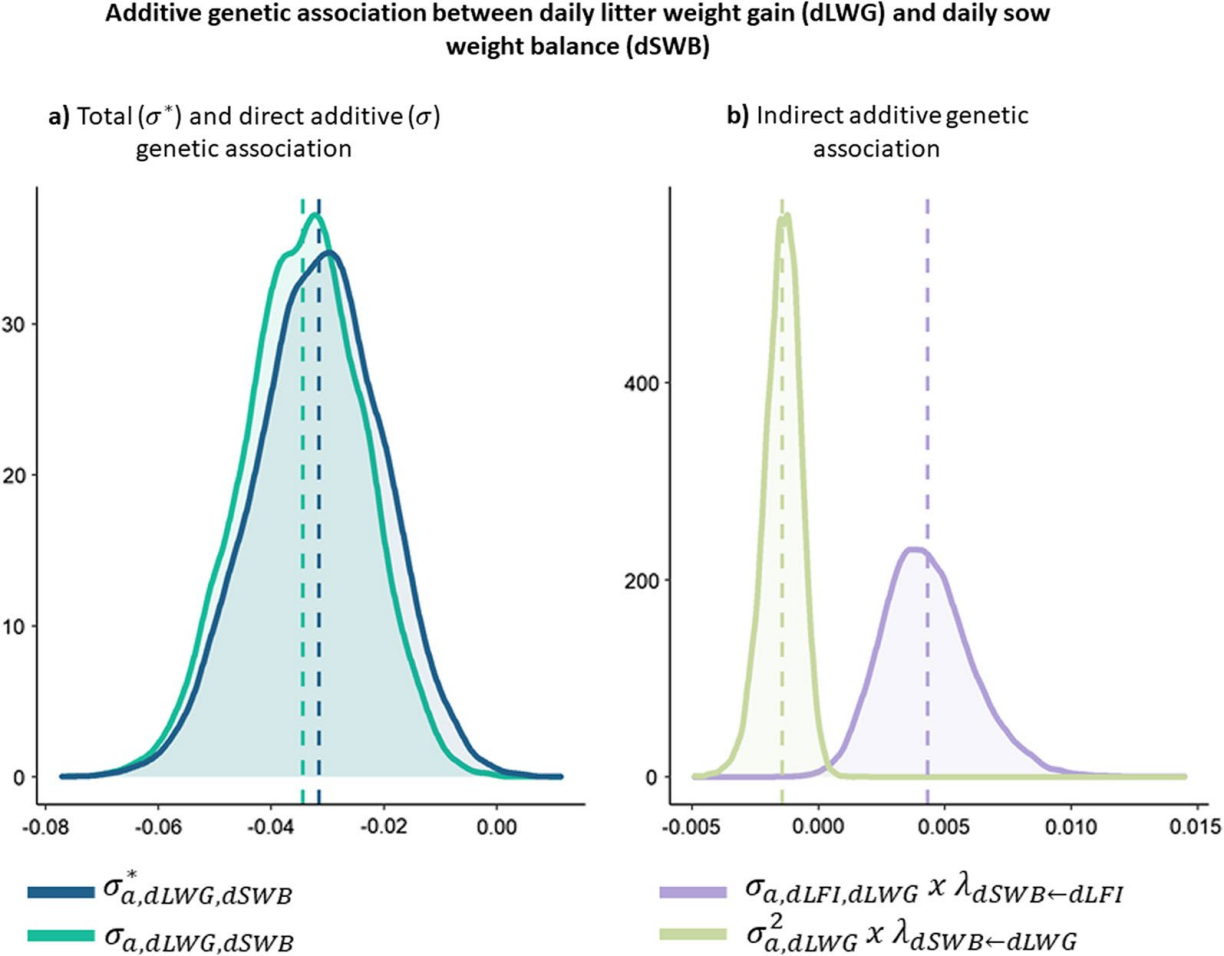
Additive genetic association between daily litter weight gain (dLWG) and daily sow weight balance (dSWB). **a** Total (${\upsigma }^{*}$) and direct additive ($\upsigma$) genetic association and **b** indirect additive genetic association

Similarly, in Fig. 3, the additive genetic associations between dLWG and dSWB are represented. Here, the total association was calculated as:

$${\upsigma }_{\mathrm{a},\mathrm{dSWB},\mathrm{dLWG}}^{*}= {\upsigma }_{\mathrm{a},\mathrm{dLFI},\mathrm{dLWG}}\times {\uplambda }_{\mathrm{dSWB}\leftarrow \mathrm{dLFI}}+{\upsigma }_{\mathrm{a},\mathrm{dLWG},\mathrm{dSWB}}+{\upsigma }_{\mathrm{a},\mathrm{dLWG}}^{2}\times {\uplambda }_{\mathrm{dSWB}\leftarrow \mathrm{dLWG}},$$

where ${\upsigma }_{\mathrm{a},\mathrm{dLWG},\mathrm{dSWB}}$ represents the direct additive genetic association between dLWG and dSWB, and ${\upsigma }_{\mathrm{a},\mathrm{dLFI},\mathrm{dLWG }}\times {\uplambda }_{\mathrm{dSWB}\leftarrow \mathrm{dLFI}}$ and ${\upsigma }_{\mathrm{a},\mathrm{dLWG }}^{2}\times {\uplambda }_{\mathrm{dSWB}\leftarrow \mathrm{dLWG}}$ are the genetic associations due to indirect effects. For these two traits, the total and direct additive genetic covariances were very similar due to the almost null contribution of the indirect effects.

**Fig. 4:**
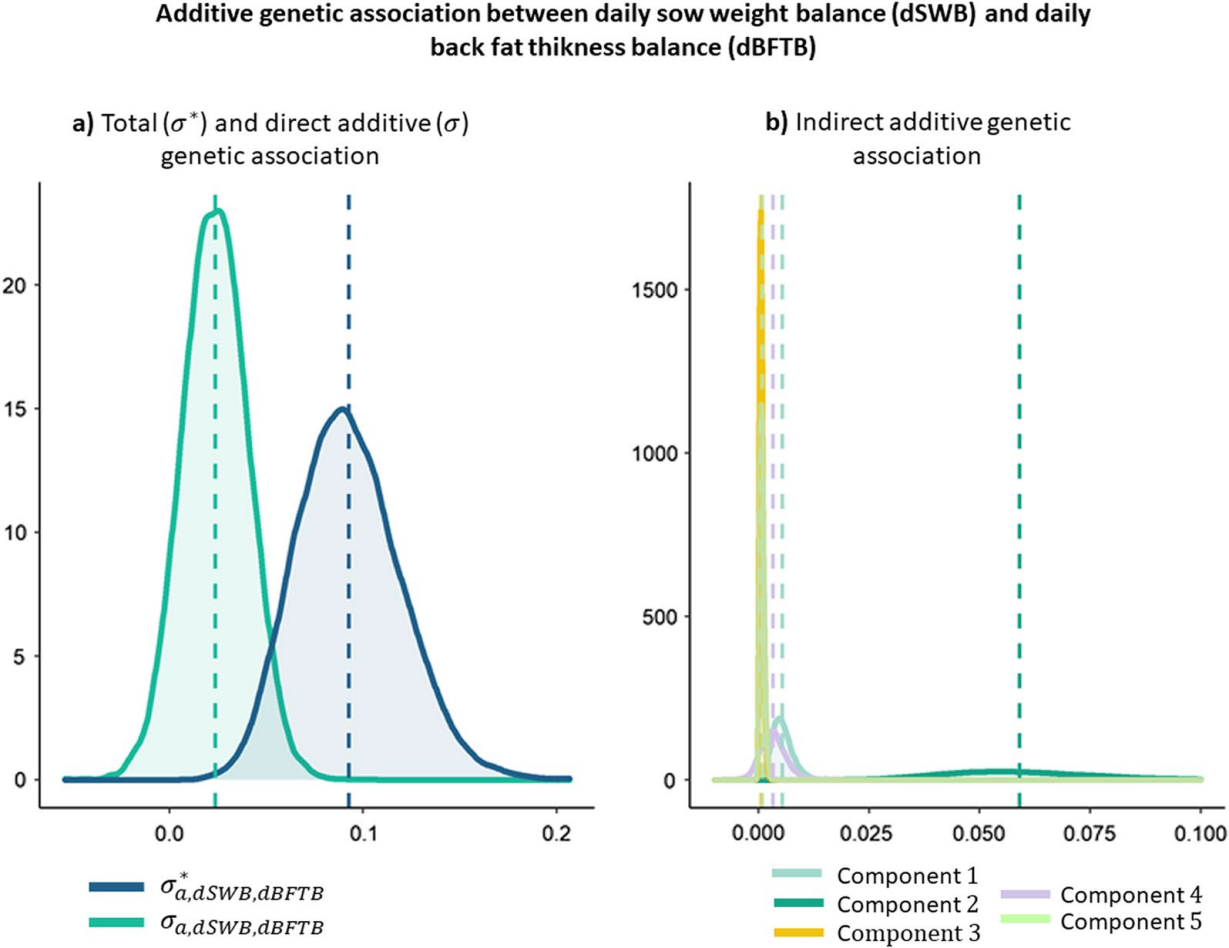
Additive genetic association between daily sow weight balance (dSWB) and daily back fat thickness balance (dBFTB). **a** Total ($${\upsigma }^{*}$$) and direct additive ($$\upsigma $$) genetic association and **b** indirect additive genetic association. $$\text{Component} \,1: \left({\uplambda }_{\mathrm{dSWB}\leftarrow \mathrm{dLFI}}\times {\upsigma }_{\mathrm{a},\mathrm{dLFI}}^{2}+{\upsigma }_{\mathrm{a},\mathrm{dLFI},\mathrm{dSWB}}+{\uplambda }_{\mathrm{dSWB}\leftarrow \mathrm{dLWG}}\times {\upsigma }_{\mathrm{a},\mathrm{dLFI},\mathrm{dLWG}}\right)\times {\uplambda }_{\mathrm{dBFTB}\leftarrow \mathrm{dSWB}}\times {\uplambda }_{\mathrm{dSWB}\leftarrow \mathrm{dLFI}},$$ $$\text{Component}\,2:\left({\uplambda }_{\mathrm{dSWB}\leftarrow \mathrm{dLFI}}\times {\upsigma }_{\mathrm{a},\mathrm{dLFI},\mathrm{dSWB}}+{\upsigma }_{\mathrm{a},\mathrm{dSWB}}^{2}+{\uplambda }_{\mathrm{dSWB}\leftarrow \mathrm{dLWG}}\times {\upsigma }_{\mathrm{a},\mathrm{dSWB},\mathrm{dLWG}}\right)\times {\uplambda }_{\mathrm{dBFTB}\leftarrow \mathrm{dSWB}},$$ $$\text{Component}\,3:\left({\uplambda }_{\mathrm{dSWB}\leftarrow \mathrm{dLFI}}\times {\upsigma }_{\mathrm{a},\mathrm{dLFI},\mathrm{dLWG}}+{\upsigma }_{\mathrm{a},\mathrm{dSWB},\mathrm{dLWG}}+{\uplambda }_{\mathrm{dSWB}\leftarrow \mathrm{dLWG}}\times {\upsigma }_{\mathrm{a},\mathrm{dLWG}}^{2}\right)\times {\uplambda }_{\mathrm{dBFTB}\leftarrow \mathrm{dSWB}}\times {\uplambda }_{\mathrm{dSWB}\leftarrow \mathrm{dLWG}},$$$$\text{Component}\,4:{\uplambda }_{\mathrm{dSWB}\leftarrow \mathrm{dLFI}}\times {\upsigma }_{\mathrm{a},\mathrm{dLFI},\mathrm{dBFTB}},$$$$\text{Component}\,5:{\uplambda }_{\mathrm{dSWB}\leftarrow \mathrm{dLWG}}\times {\upsigma }_{\mathrm{a},\mathrm{dLWG},\mathrm{dBFTB}}$$

**Fig. 5:**
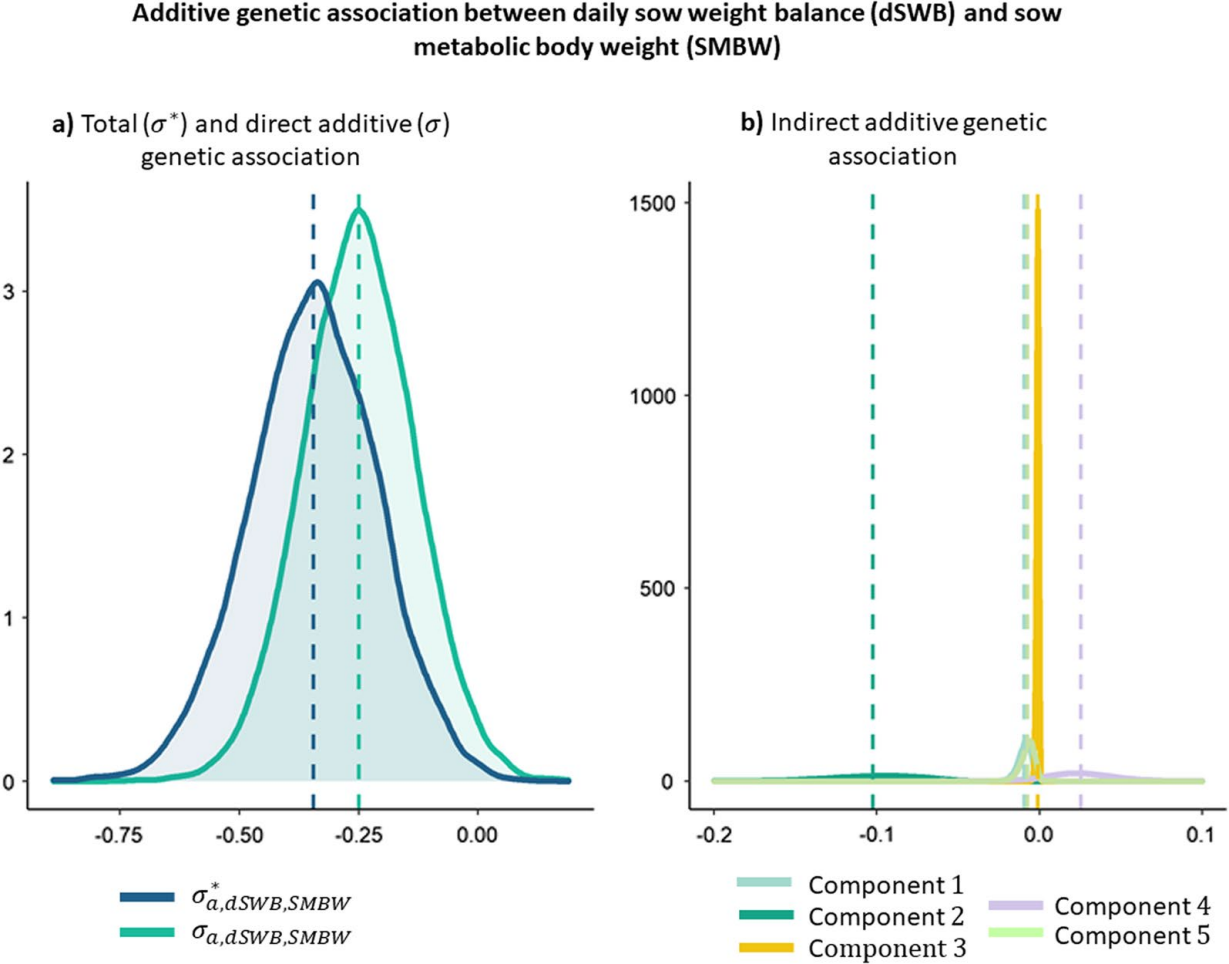
Additive genetic association between daily sow weight balance (dSWB) and sow metabolic body weight (SMBW). **a** Total (${\upsigma }^{*}$) and direct additive ($\upsigma$) genetic association and **b** indirect additive genetic association. $\text{Component}\,1:\left({\upsigma }_{\mathrm{a},\mathrm{dLFI}}^{2}\times {\uplambda }_{\mathrm{dSWB}\leftarrow \mathrm{dLFI}}+{\upsigma }_{\mathrm{a},\mathrm{dLFI},\mathrm{dSWB}}+{\upsigma }_{\mathrm{a},\mathrm{dLFI},\mathrm{dLWG}}\times {\uplambda }_{\mathrm{dSWB}\leftarrow \mathrm{dLWG}}\right){\times\uplambda }_{\mathrm{SMBW}\leftarrow \mathrm{dSWB}}\times {\uplambda }_{\mathrm{dSWB}\leftarrow \mathrm{dLFI}},$ $\text{Component}\,2:\left({\upsigma }_{\mathrm{a},\mathrm{dLFI},\mathrm{dSWB}}\times {\uplambda }_{\mathrm{dSWB}\leftarrow \mathrm{dLFI}}+{\upsigma }_{\mathrm{a},\mathrm{dSWB}}^{2}+{\upsigma }_{\mathrm{a},\mathrm{dSWB},\mathrm{dLWG}}\times {\uplambda }_{\mathrm{dSWB}\leftarrow \mathrm{dLWG}}\right)\times {\uplambda }_{\mathrm{SMBW}\leftarrow \mathrm{dSWB}},$ $\text{Component}\,3:\left({\upsigma }_{\mathrm{a},\mathrm{dLFI},\mathrm{dLWG}}\times {\uplambda }_{\mathrm{dSWB}\leftarrow \mathrm{dLFI}}+{\upsigma }_{\mathrm{a},\mathrm{dSWB},\mathrm{dLWG}}+{\upsigma }_{\mathrm{a},\mathrm{dLWG}}^{2}\times {\uplambda }_{\mathrm{dSWB}\leftarrow \mathrm{dLWG}}\right)\times {\uplambda }_{\mathrm{SMBW}\leftarrow \mathrm{dSWB}}\times {\uplambda }_{\mathrm{dSWB}\leftarrow \mathrm{dLWG}},$ $\text{Component}\,4:{\upsigma }_{\mathrm{a},\mathrm{dLFI},\mathrm{SMBW}}\times {\uplambda }_{\mathrm{dSWB}\leftarrow \mathrm{dLFI}},$ $\text{Component}\,5:{\uplambda }_{\mathrm{dSWB}\leftarrow \mathrm{dLWG}}\times {\upsigma }_{\mathrm{a},\mathrm{dLWG},\mathrm{SMBW}}$

The additive genetic associations between dSWB and dBFTB and between dSWB and SMBW are represented in Figs. 4 and 5, respectively. In these associations, multiple components contributed to the total genetic covariance. The total genetic association in the “reduced model” between dSWB and dBFTB was calculated as follows:

$${\upsigma }_{\mathrm{a},\mathrm{dSWB},\mathrm{dBFTB}}^{*}=\mathrm{Direct\, Effect}+\mathrm{Component }1+\mathrm{Component }2+\mathrm{Component }3+\mathrm{Component }4+\mathrm{Component }5,$$

where $\mathrm{Direct \,Effect}={\upsigma }_{\mathrm{a},\mathrm{dSWB},\mathrm{dLWG}}, \text{with}$ $\mathrm{Component}\,1= \left({\upsigma }_{\mathrm{a},\mathrm{dLFI}}^{2}{ \times\uplambda }_{\mathrm{dSWB}\leftarrow \mathrm{dLFI}}+{\upsigma }_{\mathrm{a},\mathrm{dLFI},\mathrm{dSWB}}+{\upsigma }_{\mathrm{a},\mathrm{dLFI},\mathrm{dLWG}}\times {\uplambda }_{\mathrm{dSWB}\leftarrow \mathrm{dLWG}}\right)\times {\uplambda }_{\mathrm{dBFTB}\leftarrow \mathrm{dSWB}}\times {\uplambda }_{\mathrm{dSWB}\leftarrow \mathrm{dLFI}},$ $\mathrm{Component }\,2= \left({\upsigma }_{\mathrm{a},\mathrm{dLFI},\mathrm{dSWB}}{ \times\uplambda }_{\mathrm{dSWB}\leftarrow \mathrm{dLFI}}+{\upsigma }_{\mathrm{a},\mathrm{dSWB}}^{2}+{\upsigma }_{\mathrm{a},\mathrm{dSWB},\mathrm{dLWG}}{\times\uplambda }_{\mathrm{dSWB}\leftarrow \mathrm{dLWG}}\right)\times {\uplambda }_{\mathrm{dBFTB}\leftarrow \mathrm{dSWB}},$ $\mathrm{Component }\,3=\left( {\upsigma }_{\mathrm{a},\mathrm{dLFI},\mathrm{dLWG}}{\times\uplambda }_{\mathrm{dSWB}\leftarrow \mathrm{dLFI}}+{\upsigma }_{\mathrm{a},\mathrm{dSWB},\mathrm{dLWG}}+{\upsigma }_{\mathrm{a},\mathrm{dLWG}}^{2}{\times\uplambda }_{\mathrm{dSWB}\leftarrow \mathrm{dLWG}}\right)\times {\uplambda }_{\mathrm{dBFTB}\leftarrow \mathrm{dSWB}}\times {\uplambda }_{\mathrm{dSWB}\leftarrow \mathrm{dLWG}},$ $\mathrm{Component }\,4={\upsigma }_{\mathrm{a},\mathrm{dLFI},\mathrm{dBFTB}}{\times\uplambda }_{\mathrm{dSWB}\leftarrow \mathrm{dLFI}},$ $\mathrm{Component }\,5={\upsigma }_{\mathrm{a},\mathrm{dLWG},\mathrm{dBFTB}}{\times\uplambda }_{\mathrm{dSWB}\leftarrow \mathrm{dLWG}}$, where the direct effect (${\upsigma }_{\mathrm{a},\mathrm{dSWB},\mathrm{dBFTB}}$) represents the direct additive genetic association between dSWB and dBFTB, and the remaining terms correspond to the contribution of different indirect effects to this covariance. The most important indirect effect was the second term of the equation (Component 2), which caused an important difference between total and direct genetic associations, while the other terms were almost negligible.

Total genetic association between dSWB and SMBW was calculated as:

$${\upsigma }_{\mathrm{a},\mathrm{dSWB},\mathrm{SMBW}}^{*}=\mathrm{ Direct\, Effect}+\mathrm{Component} \,1+\mathrm{Component}\, 2 +\mathrm{ Component } \,3+\mathrm{Component }\, 4+\mathrm{Component }\, 5,$$

where $\mathrm{Direct\, Effect}={\upsigma }_{\mathrm{a},\mathrm{dSWB},\mathrm{SMBW}},$ $\mathrm{with \,Component}\,1= \left({\upsigma }_{\mathrm{a, dLFI}}^{2}\times {\uplambda }_{\mathrm{dSWB}\leftarrow \mathrm{dLFI}}+{\upsigma }_{\mathrm{a, dLFI, dSWB}}+{\upsigma }_{\mathrm{a, dLFI, dLWG}}\times {\uplambda }_{\mathrm{dSWB}\leftarrow \mathrm{dLWG}}\right) { \times\uplambda }_{\mathrm{SMBW}\leftarrow \mathrm{dSWB}}\times {\uplambda }_{\mathrm{dSWB}\leftarrow \mathrm{dLFI}},$ $\mathrm{Component }\,2= \left({\upsigma }_{\mathrm{a, dLFI, dSWB}}\times {\uplambda }_{\mathrm{dSWB}\leftarrow \mathrm{dLFI}}+{\upsigma }_{\mathrm{a, dSWB}}^{2}+{\upsigma }_{\mathrm{a, dSWB, dLWG}}\times {\uplambda }_{\mathrm{dSWB}\leftarrow \mathrm{dLWG}}\right) \times {\uplambda }_{\mathrm{SMBW}\leftarrow \mathrm{dSWB}},$ $\mathrm{Component }\,3= \left({\upsigma }_{\mathrm{a, dLFI, dLWG}}\times {\uplambda }_{\mathrm{dSWB}\leftarrow \mathrm{dLFI}}+{\upsigma }_{\mathrm{a, dSWB, dLWG}}+{\upsigma }_{\mathrm{a, dLWG}}^{2}\times {\uplambda }_{\mathrm{dSWB}\leftarrow \mathrm{dLWG}}\right) \times {\uplambda }_{\mathrm{SMBW}\leftarrow \mathrm{dSWB}}\times {\uplambda }_{\mathrm{dSWB}\leftarrow \mathrm{dLWG}},$ $\mathrm{Component }\,4={\upsigma }_{\mathrm{a, dLFI, SMBW}}\times {\uplambda }_{\mathrm{dSWB}\leftarrow \mathrm{dLFI}},$ $\mathrm{Component}\,5={\uplambda }_{\mathrm{dSWB}\leftarrow \mathrm{dLWG}}\times {\upsigma }_{\mathrm{a, dLWG, SMBW}}$, where the direct effect $({\upsigma }_{\mathrm{a, dSWB, SMBW}}$) indicates the direct additive genetic association between dSWB and SMBW and the remaining terms correspond to the contribution of different indirect effects to this covariance. Posterior distributions of the total additive genetic covariance and of the direct additive genetic covariance were very similar to each other for these two traits because all indirect effects were small. The most important indirect contribution was the second term (Component 2).

### Responses to selection

Additional file 1: Table S3 shows the posterior mean (posterior SD) of the correlated response for each trait of the selection objective (i.e. dLFI, dSWB and dLWG) for each unit of increase in the selection index corresponding to different selection strategies. The objectives were to decrease or keep dLFI constant, to increase or keep dSWB constant, and to increase dLWG. Three selection strategies were considered: (i) selection based on an index in which the selection criterion included the measurements of dLFI, dBFTB, and dLWG; (ii) selection based on the previous index but with no information about dLFI, and (iii) selection based on information on dBFTB and dLWG when the same amount of feed was given to all sows during lactation (mimicking the effect of an external intervention that modifies the relationship between traits). Additional file 1: Table S4 shows results for the same selection strategies when information on dSWB was used instead of that on dBFTB. For all three selection strategies and regardless of whether dSWB or dBFTB was in the selection criterion, responses followed a similar pattern and, for the same economic weights, responses were the same. Table 8 shows responses to selection for the third strategy, i.e. when all the sows were fed the same amount of feed and measurements of dBFTB and dLWG were used. With this strategy, when the selection index was constrained so that there was no change in dSWB (economic weight of dSWB equal to 0), dLWG decreased by 0.09 kg/day and dLFI decreased by 0.15 kg/day. When response in dLFI was constrained to zero (economic weight of dLFI equal to 0) and dSWB had an economic weight twice that of dLWG, then dLWG decreased by 0.13 kg/day but dSWB increased by 0.14 kg/day.

**Table 8:** Responses to selection for traits in the selection objective when selection is based on daily backfat thickness and daily litter weight gain records, and sows are fed the same amount of feed

Economic weight			Correlated response		
dLFI	dSWB	dLWG	dLFI	dSWB	dLWG
− 2	1	2	− 0.11 (0.05)	− 0.11 (0.05)	0.03 (0.06)
− 2	2	1	− 0.13 (0.04)	0.09 (0.04)	− 0.17 (0.03)
− 2	1	1	− 0.16 (0.04)	0.01 (0.05)	− 0.11 (0.05)
− 2	2	2	− 0.15 (0.04)	0.03 (0.06)	− 0.12 (0.06)
− 1	1	2	0.05 (0.05)	− 0.14 (0.04)	0.17 (0.03)
− 1	2	1	− 0.06 (0.05)	0.14 (0.04)	− 0.17 (0.03)
− 1	1	1	− 0.15 (0.04)	0.03 (0.06)	− 0.12 (0.06)
− 1	2	2	0.10 (0.08)	0.06 (0.10)	0.02 (0.12)
0	1	2	0	− 0.14 (0.04)	0.13 (0.03)
0	2	1	0	0.14 (0.04)	− 0.13 (0.03)
0	1	1	0	0.07 (0.13)	− 0.06 (0.13)
− 1	0	2	0.12 (0.09)	0	0.08 (0.05)
− 2	0	1	− 0.15 (0.04)	0	− 0.09 (0.03)
− 1	0	1	− 0.15 (0.04)	0	− 0.09 (0.03)

dLFI = daily lactation feed intake (kg/day); dSWB (daily sow weight balance (kg/day); dLWG = daily litter weight gain (kg/day); dBFTB = daily back fat thickness balance (mm/day); SMBW = sow metabolic body weight (kg^0.75^)

## Discussion

Although several studies have reported genetic and phenotypic relationships between components of lactation feed efficiency [7], to our knowledge, there is no study on the phenotypic causal relationships between these components. We investigated the causal structure among five traits that are involved in LFE and estimated the magnitude of the putative causal effects in this causal structure using data from a population of pigs selected for RFI during growth. The causal structure between components of FE was previously studied by Wu et al. [15] in dairy cattle, but they defined the causal structure by assuming that energy sinks affect FI and that there are no causal relationships between energy sinks. This model means a causal interpretation of RFI by phenotypic recursiveness between FI and energy sinks, which allows the estimation of breeding values and genetic parameters for RFI directly (i.e. with no previous estimation of RFI).

Structural equation models describe the causal relationships that are present in a biological system, but it requires the specification of a potential causal structure underlying it. The IC algorithm is one of many available strategies to search for a network structure that is compatible with the joint probability distribution of the traits considered. However, the IC algorithm requires the assumption that there are no simultaneous relationships (i.e., feedbacks) between the traits. In an IC search, unobserved correlated genetic or environmental effects that are usually present for livestock production traits could be confounded with causal relationships. To avoid this problem, we implemented the IC algorithm on the joint distribution of phenotypes conditional to unobservable genetic and environmental effects, as proposed by Valente et al. [10], using a Bayesian approach. However, for some sets of traits, it may not be possible to find the edges and the causal directions using only information from the data. In this situation, biological prior knowledge can be incorporated to complete the causal structure specification. Thus, for example, Chitakasempornkul et al. [26] incorporated temporal information to fully orient the network that had an unresolved connection between two traits based on the IC algorithm. However, this extra information could be subjective and does not guarantee causality. The causal structure that is returned by the IC algorithm can also change depending on the HPD interval used [27]. In our analysis, the data-driven IC algorithm yielded a fully directed acyclic graph using HPD_95%_ intervals. When 70, 80, and 90% HPD intervals were used, the IC algorithm returned the same structure but with an additional edge between dLFI and dLWG, but without directions. Therefore, we assumed the causal structure returned by the IC algorithm using HPD_95%_ intervals as the causal structure for the SEM analysis. The resulting estimates indicated that an increase in dLFI would increase dSWB, while an increase in dLWG would decrease dSWB and, in turn, an increase in dSWB would increase dBFTB while decreasing SMBW (Fig. 1).

After implementing the SEM, the sign of the structural coefficients agreed with the sign of the residual correlations obtained with the MTAM. Estimates of the structural coefficients express the strength of each causal link between traits, which is essential to quantify the component of every element of the variance–covariance matrices that results from indirect sources of (co)variation. Estimates of variances and of ratios of variances, such as heritability, based on a SEM (direct effects) or a MTAM (total effects) are expected to be the same for the upstream traits (i.e., traits that are not affected by other traits in the causal network). In our study, this principle was fulfilled for dLFI but not for dLWG because of the unexpected difference in estimates of the variance of permanent environmental effects, which was equal to 0.008 (0.005) based on the SEM and 0.02 (0.006) based on the MTAM. This difference could be due to the constraint imposed on the residual covariance which is set to zero in the SEM but not in the MTAM. For traits that are affected by other traits in the causal network, indirect effects may contribute to variances and ratios of variances, resulting in differences between estimates based on the MTAM and estimates based on the SEM. In our study, dBFTB was the most affected trait with the posterior mean (posterior SD) of the total additive genetic variance being 0.16 (0.03), while the additive genetic variance of direct effects was 0.07 (0.02), which resulted in relevant (although not statistically different) differences between estimates of total and direct heritability for this trait (0.18 (0.04) and 0.12 (0.03), respectively). Therefore, the total genetic effects for this trait were largely due to indirect effects that originated from the upstream traits, mainly dSWB.

Knowing which proportion of the relationship between two traits is due to direct and indirect effects is important to design new selection strategies based on external interventions for a trait without the need to perform an experiment to obtain covariance estimates with those interventions. Such external interventions are those that can change or block a causal relationship between traits (e.g. the practice of cesarians removes the effect of gestation length or fetus weight on calving difficulty) or that control the phenotypic value of one trait, e.g. by holding it constant (e.g. cross-fostering, or feed restriction to a fixed amount of feed), as was the case for one of our proposed selection strategies.

In the specific case of the relationship between dLFI and dSWB, the causal structure indicates that dLFI positively affects dSWB, with an increase in dSWB of 0.12 kg/day for every 1 kg/day increase in dLFI. The mainly contribution of indirect effects correspond to the term ${\upsigma }_{\mathrm{a, dLFI}}^{2}\times {\uplambda }_{\mathrm{dSWB}\leftarrow \mathrm{dLFI}}$ since the term ${\upsigma }_{\mathrm{a, dLFI},\mathrm{dLWG}}\times {\uplambda }_{\mathrm{dSWB}\leftarrow \mathrm{dLWG}}$ had almost no contribution (Fig. 3).

The causal effect of dLWG on dSWB was negative and the lowest in magnitude compared to all other causal effects among the analyzed traits. For every 1 kg/day increase in dLWG, dSWB is expected to decrease by 0.03 kg/day. Piles et al. [7] also found a negative residual correlation between dLWG and dSWB in a Duroc population of pigs. This negative effect can be explained by the sow needing to mobilize body reserves to meet the increased milk demands of the piglets as a result of increased growth. Selection to increase dLWG is expected to result in a negative correlated response in dSWB since the total genetic correlation between dLWG and dSWB is low to moderate and negative (posterior mean − 0.37).

Previous studies have demonstrated the influence of lactation feed intake on litter weight gain. Eissen et al. [28] showed that litter weight gain increased as feed intake of the sows increased. Moreover, Hawe et al. [29] estimated the effect of increasing sow feed intake on piglet growth and showed that better sow feeding during lactation can increase milk yields and piglet weaning weights. In our analyses using the IC algorithm, we observed a non-directed edge between dLFI and dLWG, which disappeared when HPD_95%_ were used instead of HPD intervals with smaller levels of probability content. Given the published information regarding the effect of dLFI on dLWG, and given our results using 70, 80, and 90% HPD intervals, we implemented a SEM that also included the corresponding structural coefficient between dLFI and dLWG. We found that this did not affect estimates of variance components of the model, which were very close to those obtained with the model that did not include this structural coefficient (results not shown), and that the estimate of the structural coefficient indicated a positive but small effect of dLFI on dLWG (0.03 kg/day for each 1 kg/day increase in dLFI).

Based on the posterior means of the structural coefficients, the most important causal effect was a negative effect of dSWB on SMBW. The estimate of the corresponding structural coefficient (${\uplambda }_{\mathrm{SMBW}\leftarrow \mathrm{dSWB}}$) indicated that for each kg/day increase in dSWB, SMBW is expected to decrease by + 1.22 kg/day. Abdalla et al. [14] also found an edge between body weight gain and metabolic mid-weight in turkeys, but in contrast to our results, the latter trait had a positive effect on the former in their analyses, which means that increasing energy for maintenance would lead to an increase in weight. However it should be noted that this study has important differences with ours, not only regarding the species and the set of analyzed traits but also the method of analysis, which might explain this difference. According to our results, selection for increased dSWB is expected to reduce requirements for maintenance given the low to moderate total genetic correlation between those traits (− 0.28). Many indirect effects contribute to this covariance but the most important were those involved in the term $\left({\upsigma }_{\mathrm{a, dLFI},\mathrm{dSWB}}\times {\uplambda }_{\mathrm{dSWB}\leftarrow \mathrm{dLFI}}+{\upsigma }_{\mathrm{a, dSWB}}^{2}+{\upsigma }_{\mathrm{a, dSWB, dLWG}}\times {\uplambda }_{\mathrm{dSWB}\leftarrow \mathrm{dLWG}}\right)\times {\uplambda }_{\mathrm{SMBW}\leftarrow \mathrm{dSWB}}$, for which the structural coefficient ${\lambda }_{SMBW\leftarrow dSWB}$ was the most relevant (− 1.22). The remaining indirect effects were very small and hardly contributed to the total genetic covariance.

Daily backfat thickness balance is a component of dSWB, since it is a measure for a specific tissue among all those that compose the body. This trait is easier to measure on a nucleus farm than dSWB because it can be done in situ. Therefore, it is interesting to determine if it can be used as a good marker of the body condition of a sow for selection instead of dSWB. The IC algorithm found an effect of dSWB on dBFTB and the structural coefficient estimated for this causal relationship in the SEM indicated that an increase of 1 kg/day in dSWB is expected to increase dBFTB by 0.070 mm/day. Our results showed that dSWB and dBFTB are highly correlated, with the posterior mean of the total genetic correlation being equal to 0.69. However, the direct genetic correlation was 0.31, which indicates that indirect effects exerted by the other traits are quite relevant in this relationship. Many indirect effects contributed to this correlation but the most important was $\left({\uplambda }_{\mathrm{dSWB}\leftarrow \mathrm{dLFI}}\times {\upsigma }_{\mathrm{a, dLFI},\mathrm{dSWB}}+{\upsigma }_{\mathrm{a, dSWB}}^{2}+{\uplambda }_{\mathrm{dSWB}\leftarrow \mathrm{dLWG}}\times {\upsigma }_{\mathrm{a, dSWB, dLWG}}\right)\times {\uplambda }_{\mathrm{dBFTB}\leftarrow \mathrm{dSWB}}$.

Measurement of individual feed intake is expensive and labor-intensive even when electronic feeders are used. Given the close relationship between dLFI and dSWB, it is relevant to assess whether a measure of dSWB provides enough information on dLFI to allow effective selection for components of LFE without LFI recording. Likewise, since dBFTB is easier to record than dSWB and both traits are strongly correlated, assessing whether dBFTB could replace dSWB in the selection index, is relevant. Correlated responses to selection in the targeted traits were little affected by whether measurements of dBFTB or dSWB were included, which indicates that dBFTB could replace dSWB in a selection criterion. Responses to selection following the former two strategies showed that selection without phenotypic information on dLFI is possible since correlated responses were little affected by the lack of dLFI data. However, given the covariance structure for all traits involved in LFE, it is unlikely that a reduction in dLFI while increasing sow productivity and no decline in sow body condition can be achieved. Piles et al. [7] found that selection for restricted RFI (i.e., RFI estimated from genetic regression of FI on production traits instead of from phenotypic regression [8]) is not expected to be effective because of the low genetic variation of this trait. Restricted residual feed intake is equivalent to a restricted selection index in which production traits (in our case, dSWB or dBFT and dLWG) are held constant. This definition of FE guarantees null genetic correlations of RFI with production traits and thus null correlated responses in these traits when selecting for RFI.

Providing the same amount of feed to all sows during lactation would remove a source of individual variation in LFE. In this case, variation in efficiency would be due to variation in the other traits, which are easier to measure than LFI. This strategy was successfully implemented in growing pigs by Nguyen et al. [9] for improving feed efficiency during growth but, in that selection experiment, animals were fed a fixed but restricted ration of approximately 80% of averaged ad libitum intake over 6 weeks, which provided the conditions to better observe differences in FE among individuals. In our study, by keeping the amount of feed provided to all the sows constant and including only dSWB or dBFTB and dLWG in the selection criterion, the same pattern of responses was obtained as with selection strategies in which this management practice was not implemented. In addition, the magnitudes of the responses in each trait of the selection objective for the same economic weights were not significantly different from each other. Therefore, this management strategy does not seem to offer an advantage over the others and is not recommended.

## Conclusions

Knowing the causal relationships among traits involved in LFE allows predicting the outcome of external interventions in a trait and how it is expected to affect responses to selection in a breeding program. For the first time, we have deciphered the causal structure among the main components of LFE and estimated the magnitude of their associations in a pig population. The results indicate that dLFI and dLWG affect dSWB, which, in turn, affects dBFTB and SMBW. Indirect effects are particularly relevant for the genetic variance of dBFTB and SMBW, which are indirectly affected by all other traits through dSWB. The contribution of indirect effects to the total genetic covariance between traits is most important in the relationship between dSWB and dBFTB. Responses to selection in the objective traits were, however, little affected by the different selection strategies evaluated, including not recording LFI or imposing the external management practice of restricted feeding, and no strategy offered a clear advantage over the others. In addition, it appears difficult to reduce dLFI while increasing sow productivity without impairing sow body condition.

## Supplementary Information

**Additional file 1: Table S1.** Posterior means of permanent correlations and 95% highest posterior density (HPD_95%_) intervals among traits based on the multiple trait animal model and on the structural equation model (SEM). **Table S2.** Posterior means of contemporary group correlations and 95% highest posterior density (HPD_95%_) intervals among traits based on the multiple trait animal model and on the structural equation model (SEM). **Table S3.** Posterior means (posterior SD) of correlated response to selection of each trait of the selection objective. **Table S4.** Posterior means (posterior SD) of correlated response to selection of each trait of the selection objective.

## Data Availability

The datasets during and/or analyzed during the current study are available from the corresponding author upon reasonable request.
