# Supplementary material for: Analysis of the causal structure of traits involved in sow lactation feed efficiency
Source: Genet Sel Evol. 2022 Jul 26;54:53. doi: 10.1186/s12711-022-00744-4 (PMC9327305; doi:10.1186/s12711-022-00744-4)
Supplement: Supplementary file 1 — Additional file 1: Table S1. Posterior means of permanent correlations and 95% highest posterior density (HPD95%) intervals among traits based on the multiple trait animal model and on the structural equation model (SEM). Table S2. Posterior means of contemporary group correlations and 95% highest posterior density (HPD95%) intervals among traits based on the multiple trait animal model and on the structural equation model (SEM). Table S3. Posterior means (posterior SD) of correlated response to selection of each trait of the selection objective. Table S4. Posterior means (posterior SD) of correlated response to selection of each trait of the selection objective. [file 12711_2022_744_MOESM1_ESM.docx]

**Additional file 1 Table S1**

**Posterior means of permanent correlations and 95% highest posterior density (HPD_95%_) intervals among traits based on the multiple trait animal model (MTAM) and on the structural equation model (SEM)**

| **^2^Trait** | **^2^Trait** | **^1^MTAM** | | **^1^SEM** | |
| --- | --- | --- | --- | --- | --- |
|  |  | **Posterior Mean** | **HPD_95%_** | **Posterior Mean** | **HPD_95%_** |
| dLFI | dSWB | 0.30 | [-0.18,0.74] | 0.61 | [0.27,0.93] |
|  | dLWG | 0.56 | [-0.03,0.97] | 0.55 | [0.06,0.93] |
|  | dBFTB | 0.29 | [-0.23,0.80] | 0.31 | [-0.20,0.80] |
|  | SMBW | -0.39 | [-0.80,0.07] | 0.04 | [-0.33,0.37] |
| dSWB | dLWG | -0.09 | [-0.58,0.39] | -0.21 | [-0.80,0.57] |
|  | dBFTB | 0.69 | [0.40,0.93] | 0.22 | [-0.31,0.75] |
|  | SMBW | 0.22 | [-0.12,0.57] | 0.39 | [0.05,0.72] |
| dLWG | dBFTB | 0.07 | [-0.49,0.64] | 0.12 | [-0.42,0.70] |
|  | SMBW | -0.14 | [-0.55,0.25] | -0.36 | [-0.76,0.07] |
| dBFTB | SMBW | -0.14 | [-0.40,0.14] | -0.06 | [-0.42,0.31] |

^1^MTAM = Multiple trait animal model; SEM = Structural equation model

^2^dLFI = daily lactation feed intake (kg/day); dSWB (daily sow weight balance (kg/day); dLWG = daily litter weight gain (kg/day); dBFTB = daily back fat thickness balance (mm/day); SMBW = sow metabolic body weight (kg^0.75^).

**Additional file 1 Table S2**

**Posterior means of contemporary group correlations and 95% highest posterior density (HPD_95%_) intervals among traits based on the multiple trait animal model (MTAM) and on the structural equation model (SEM)**

| **^2^Trait** | **^2^Trait** | **^1^MTAM** | | **^1^SEM** | |
| --- | --- | --- | --- | --- | --- |
|  |  | **Posterior Mean** | **HPD_95%_** | **Posterior Mean** | **HPD_95%_** |
| dLFI | dSWB | 0.30 | [0.11,0.48] | 0.16 | [-0.06,0.38] |
|  | dLWG | 0.19 | [-0.04,0.40] | 0.20 | [-0.02,0.42] |
|  | dBFTB | 0.18 | [-0.07,0.43] | -0.02 | [-0.30,0.24] |
|  | SMBW | -0.32 | [-0.51,-0.12] | -0.29 | [-0.49,-0.09] |
| dSWB | dLWG | 0.08 | [-0.15,0.31] | -0.02 | [-0.25,0.21] |
|  | dBFTB | 0.56 | [0.34,0.76] | 0.05 | [-0.37,0.46] |
|  | SMBW | -0.36 | [-0.54,-0.16] | -0.16 | [-0.39,0.06] |
| dLWG | dBFTB | -0.10 | [-0.38,0.18] | -0.19 | [-0.46,0.10] |
|  | SMBW | -0.01 | [-0.25,0.23] | 0.01 | [-0.23,0.24] |
| dBFTB | SMBW | -0.33 | [-0.60,-0.04] | -0.22 | [-0.55,0.11] |

^1^MTAM = Multiple trait animal model; SEM = Structural equation model

^2^dLFI = daily lactation feed intake (kg/day); dSWB (daily sow weight balance (kg/day); dLWG = daily litter weight gain (kg/day); dBFTB = daily back fat thickness balance (mm/day); SMBW = sow metabolic body weight.

**Additional file 1 Table S3**

**Posterior means (posterior SD) of correlated response to selection of each trait of the selection objective**

|  | **^1^Traits in the selection objective** | | | | | | |
| --- | --- | --- | --- | --- | --- | --- | --- |
|  | **Economic weight** | | | | **Correlated response** | | |
|  |  |  |  |  | |  |  |
| **Traits in the selection criterion**^1^ | dLFI | dSWB | dLWG | dLFI | | dSWB | dLWG |
| dLFI, dBFTB, dLWG | -2 | 1 | 2 | -0.20 (0.06) | | -0.14 (0.05) | 0.01 (0.05) |
| dLFI, dBFTB, dLWG | -2 | 2 | 1 | -0.19 (0.06) | | 0.04 (0.06) | -0.16 (0.03) |
| dLFI, dBFTB, dLWG | -2 | 1 | 1 | -0.24 (0.05) | | -0.06 (0.05) | -0.09 (0.04) |
| dLFI, dBFTB, dLWG | -2 | 2 | 2 | -0.23 (0.05) | | -0.04 (0.06) | -0.10 (0.05) |
| dLFI, dBFTB, dLWG | -1 | 1 | 2 | 0.008 (0.07) | | -0.16 (0.04) | 0.16 (0.04) |
| dLFI, dBFTB, dLWG | -1 | 2 | 1 | -0.02 (0.07) | | 0.16 (0.04) | -0.16 (0.04) |
| dLFI, dBFTB, dLWG | -1 | 1 | 1 | -0.23 (0.05) | | -0.04 (0.06) | -0.10 (0.05) |
| dLFI, dBFTB, dLWG | -1 | 2 | 2 | 0.18 (0.08) | | 0.10 (0.07) | 0.04 (0.08) |
| dLFI, dBFTB, dLWG | 0 | 1 | 2 | 0 | | -0.15 (0.04) | 0.15 (0.03) |
| dLFI, dBFTB, dLWG | 0 | 2 | 1 | 0 | | 0.16 (0.04) | -0.15 (0.03) |
| dLFI, dBFTB, dLWG | 0 | 1 | 1 | 0 | | 0.07 (0.10) | -0.05 (0.10) |
| dLFI, dBFTB, dLWG | 0 | 2 | 2 | 0 | | 0.07 (0.10) | -0.05 (0.10) |
| dLFI, dBFTB, dLWG | -1 | 0 | 2 | 0.17 (0.07) | | 0 | 0.19 (0.04) |
| dLFI, dBFTB, dLWG | -2 | 0 | 1 | -0.21 (0.04) | | 0 | -0.13 (0.03) |
| dLFI, dBFTB, dLWG | -1 | 0 | 1 | -0.20 (0.04) | | 0 | -0.13 (0.03) |
| dLFI, dBFTB, dLWG | -2 | 0 | 2 | -0.20 (0.04) | | 0 | -0.13 (0.03) |
| dBFTB, dLWG | -2 | 1 | 2 | -0.12 (0.05) | | -0.11 (0.05) | 0.04 (0.06) |
| dBFTB, dLWG | -2 | 2 | 1 | -0.14 (0.05) | | 0.08 (0.05) | -0.16 (0.03) |
| dBFTB, dLWG | -2 | 1 | 1 | -0.17 (0.04) | | -0.003 (0.05) | -0.09 (0.05) |
| dBFTB, dLWG | -2 | 2 | 2 | -0.16 (0.04) | | 0.006 (0.06) | -0.10 (0.05) |
| dBFTB, dLWG | -1 | 1 | 2 | 0.03 (0.06) | | -0.15 (0.04) | 0.16 (0.03) |
| dBFTB, dLWG | -1 | 2 | 1 | -0.06 (0.06) | | 0.14 (0.04) | -0.17 (0.03) |
| dBFTB, dLWG | -1 | 1 | 1 | -0.16 (0.04) | | 0.006 (0.06) | -0.10 (0.05) |
| dBFTB, dLWG | -1 | 2 | 2 | 0.10 (0.10) | | 0.04 (0.11) | 0.03 (0.13) |
| dBFTB, dLWG | 0 | 1 | 2 | 0 | | -0.15 (0.04) | 0.15 (0.03) |
| dBFTB, dLWG | 0 | 2 | 1 | 0 | | 0.15 (0.04) | -0.14 (0.03) |
| dBFTB, dLWG | 0 | 1 | 1 | 0 | | 0.04 (0.14) | -0.02 (0.14) |
| dBFTB, dLWG | 0 | 2 | 2 | 0 | | 0.04 (0.14) | -0.02 (0.14) |
| dBFTB, dLWG | -1 | 0 | 2 | 0.11 (0.12) | | 0 | 0.07 (0.07) |
| dBFTB, dLWG | -2 | 0 | 1 | -0.17 (0.04) | | 0 | -0.09 (0.03) |
| dBFTB, dLWG | -1 | 0 | 1 | -0.17 (0.04) | | 0 | -0.09 (0.03) |
| dBFTB, dLWG | -2 | 0 | 2 | -0.17 (0.04) | | 0 | -0.09 (0.03) |
| dBFTB, dLWG, dLFI=restricted | -2 | 1 | 2 | -0.11 (0.05) | | -0.11 (0.05) | 0.03 (0.06) |
| dBFTB, dLWG, dLFI= restricted | -2 | 2 | 1 | -0.13 (0.04) | | 0.09 (0.04) | -0.17 (0.03) |
| dBFTB, dLWG, dLFI= restricted | -2 | 1 | 1 | -0.16 (0.04) | | 0.01 (0.05) | -0.11 (0.05) |
| dBFTB, dLWG, dLFI= restricted | -2 | 2 | 2 | -0.15 (0.04) | | 0.03 (0.06) | -0.12 (0.06) |
| dBFTB, dLWG, dLFI= restricted | -1 | 1 | 2 | 0.05 (0.05) | | -0.14 (0.04) | 0.17 (0.03) |
| dBFTB, dLWG, dLFI= restricted | -1 | 2 | 1 | -0.06 (0.05) | | 0.14 (0.04) | -0.17 (0.03) |
| dBFTB, dLWG, dLFI= restricted | -1 | 1 | 1 | -0.15 (0.04) | | 0.03 (0.06) | -0.12 (0.06) |
| dBFTB, dLWG, dLFI= restricted | -1 | 2 | 2 | 0.10 (0.08) | | 0.06 (0.10) | 0.02 (0.12) |
| dBFTB, dLWG, dLFI= restricted | 0 | 1 | 2 | 0 | | -0.14 (0.04) | 0.13 (0.03) |
| dBFTB, dLWG, dLFI= restricted | 0 | 2 | 1 | 0 | | 0.14 (0.04) | -0.13 (0.03) |
| dBFTB, dLWG, dLFI= restricted | 0 | 1 | 1 | 0 | | 0.07 (0.13) | -0.06 (0.13) |
| dBFTB, dLWG, dLFI= restricted | 0 | 2 | 2 | 0 | | 0.07 (0.13) | -0.06 (0.13) |
| dBFTB, dLWG, dLFI= restricted | -1 | 0 | 2 | 0.12 (0.09) | | 0 | 0.08 (0.05) |
| dBFTB, dLWG, dLFI= restricted | -2 | 0 | 1 | -0.15 (0.04) | | 0 | -0.09 (0.03) |
| dBFTB, dLWG, dLFI= restricted | -1 | 0 | 1 | -0.15 (0.04) | | 0 | -0.09 (0.03) |
| dBFTB, dLWG, dLFI= restricted | -2 | 0 | 2 | -0.15 (0.04) | | 0 | -0.09 (0.03) |

^1^dLFI = daily lactation feed intake (kg/day); dSWB (daily sow weight balance (kg/day); dLWG = daily litter weight gain (kg/day); dBFTB = daily back fat thickness balance (mm/day); SMBW = sow metabolic body weight (kg^0.75^).

**Additional file 1 Table S4**

**Posterior means (posterior SD) of correlated response to selection of each trait of the selection objective**

|  | **^1^Traits in the selection objective** | | | | | | |
| --- | --- | --- | --- | --- | --- | --- | --- |
|  | **Economic weight** | | | | **Correlated response** | | |
|  |  |  |  |  | |  |  |
| **Traits in the selection criterion**^1^ | dLFI | dSWB | dLWG | dLFI | | dSWB | dLWG |
| dLFI, dSWB, dLWG | -2 | 1 | 2 | -0.20 (-0.06) | | -0.16 (0.05) | 0.006 (0.05) |
| dLFI, dSWB, dLWG | -2 | 2 | 1 | -0.17 (0.06) | | 0.08 (0.06) | -0.17 (0.03) |
| dLFI, dSWB, dLWG | -2 | 1 | 1 | -0.23 (0.05) | | -0.06 (0.06) | -0.10 (0.04) |
| dLFI, dSWB, dLWG | -2 | 2 | 2 | -0.20 (0.07) | | 0.01 (0.08) | -0.13 (0.05) |
| dLFI, dSWB, dLWG | -1 | 1 | 2 | 0.03 (0.07) | | -0.16 (0.05) | 0.16 (0.03) |
| dLFI, dSWB, dLWG | -1 | 2 | 1 | 0.01 (0.07) | | -0.20 (0.05) | -0.14 (0.04 |
| dLFI, dSWB, dLWG | -1 | 1 | 1 | -0.20 (0.06) | | 0.01 (0.08) | -0.13 (0.05) |
| dLFI, dSWB, dLWG | -1 | 2 | 2 | 0.18 (0.07) | | 0.16 (0.06) | -0.006 (0.07) |
| dLFI, dSWB, dLWG | 0 | 1 | 2 | 0 | | -0.17 (0.04) | 0.15 (0.03) |
| dLFI, dSWB, dLWG | 0 | 2 | 1 | 0 | | 0.19 (0.04) | -0.15 (0.03) |
| dLFI, dSWB, dLWG | 0 | 1 | 1 | 0 | | 0.15 (0.07) | -0.10 (0.06) |
| dLFI, dSWB, dLWG | 0 | 2 | 2 | 0 | | 0.15 (0.07) | -0.10 (0.06) |
| dLFI, dSWB, dLWG | -1 | 0 | 2 | 0.17 (0.06) | | 0 | 0.12 (0.04) |
| dLFI, dSWB, dLWG | -2 | 0 | 1 | -0.21 (0.04) | | 0 | -0.13 (0.03) |
| dLFI, dSWB, dLWG | -1 | 0 | 1 | -0.20 (0.05) | | 0 | -0.12 (0.03) |
| dLFI, dSWB, dLWG | -2 | 0 | 2 | -0.20 (0.05) | | 0 | -0.12 (0.03) |
| dSWB, dLWG | -2 | 1 | 2 | -0.14 (0.05) | | -0.17 (0.05) | 0.04 (0.06) |
| dSWB, dLWG | -2 | 2 | 1 | -0.11 (0.06) | | 0.12 (0.06) | -0.17 (0.03) |
| dSWB, dLWG | -2 | 1 | 1 | -0.19 (0.04) | | -0.03 (0.07) | -0.10 (0.05) |
| dSWB, dLWG | -2 | 2 | 2 | -0.14 (0.06) | | 0.07 (0.09) | -0.15 (0.05) |
| dSWB, dLWG | -1 | 1 | 2 | 0.06 (0.06) | | -0.16 (0.05) | 0.17 (0.03) |
| dSWB, dLWG | -1 | 2 | 1 | 0.02 (0.06) | | 0.20 (0.05) | -0.15 (0.04) |
| dSWB, dLWG | -1 | 1 | 1 | -0.14 (0.06) | | 0.07 (0.09) | -0.15 (0.05) |
| dSWB, dLWG | -1 | 2 | 2 | 0.15 (0.06) | | 0.16 (0.06) | -0.01 (0.07) |
| dSWB, dLWG | 0 | 1 | 2 | 0 | | -0.18 (0.04) | 0.15 (0.03) |
| dSWB, dLWG | 0 | 2 | 1 | 0 | | 0.19 (0.04) | -0.15 (0.03) |
| dSWB, dLWG | 0 | 1 | 1 | 0 | | 0.17 (0.08) | -0.13 (0.07) |
| dSWB, dLWG | 0 | 2 | 2 | 0 | | 0.17 (0.08) | -0.13 (0.07) |
| dSWB, dLWG | -1 | 0 | 2 | 0.17 (0.04) | | 0 | 0.12 (0.03) |
| dSWB, dLWG | -2 | 0 | 1 | -0.17 (0.04) | | 0 | -0.12 (0.03) |
| dSWB, dLWG | -1 | 0 | 1 | -0.17 (0.04) | | 0 | -0.12 (0.03) |
| dSWB, dLWG | -2 | 0 | 2 | -0.17 (0.04) | | 0 | -0.12 (0.03) |
| dSWB, dLWG, dLFI= restricted | -2 | 1 | 2 | -0.11 (0.06) | | -0.16 (0.06) | 0.03 (0.08) |
| dSWB, dLWG, dLFI= restricted | -2 | 2 | 1 | -0.08 (0.05) | | 0.15 (0.05) | -0.18 (0.03) |
| dSWB, dLWG, dLFI= restricted | -2 | 1 | 1 | -0.16 (0.04) | | 0.01 (0.07) | -0.13 (0.05) |
| dSWB, dLWG, dLFI= restricted | -2 | 2 | 2 | -0.07 (0.06) | | 0.15 (0.07) | -0.17 (0.04) |
| dSWB, dLWG, dLFI= restricted | -1 | 1 | 2 | 0.10 (0.06) | | -0.12 (0.06) | 0.17 (0.04) |
| dSWB, dLWG, dLFI= restricted | -1 | 2 | 1 | 0.007 (0.06) | | 0.20 (0.04) | -0.14 (0.04) |
| dSWB, dLWG, dLFI= restricted | -1 | 1 | 1 | -0.07 (0.06) | | 0.15 (0.07) | -0.17 (0.04) |
| dSWB, dLWG, dLFI= restricted | -1 | 2 | 2 | 0.12 (0.05) | | 0.16 (0.06) | -0.02 (0.06) |
| dSWB, dLWG, dLFI= restricted | 0 | 1 | 2 | 0 | | -0.19 (0.05) | 0.14 (0.04) |
| dSWB, dLWG, dLFI= restricted | 0 | 2 | 1 | 0 | | 0.19 (0.04) | -0.14 (0.04) |
| dSWB, dLWG, dLFI= restricted | 0 | 1 | 1 | 0 | | 0.19 (0.05) | -0.13 (0.04) |
| dSWB, dLWG, dLFI= restricted | 0 | 2 | 2 | 0 | | 0.19 (0.05) | -0.13 (0.04) |
| dSWB, dLWG, dLFI= restricted | -1 | 0 | 2 | 0.15 (0.04) | | 0 | 0.12 (0.03) |
| dSWB, dLWG, dLFI= restricted | -2 | 0 | 1 | -0.15 (0.04) | | 0 | -0.12 (0.03) |
| dSWB, dLWG, dLFI= restricted | -1 | 0 | 1 | -0.15 (0.05) | | 0 | -0.16 (0.04) |
| dSWB, dLWG, dLFI= restricted | -2 | 0 | 2 | -0.15 (0.05) | | 0 | -0.16 (0.04) |

^1^dLFI = daily lactation feed intake (kg/day); dSWB (daily sow weight balance (kg/day); dLWG = daily litter weight gain (kg/day); dBFTB = daily back fat thickness balance (mm/day); SMBW = sow metabolic body weight (kg^0.75^).

Total additive genetic (co)variance ($\sigma_{a,x,y}^{*}$) among the main components of lactation feed efficiency is represented as follow:

$\left( \begin{matrix} \sigma_{a,dLFI}^{2*} & \sigma_{a,dLFI,dSWB}^{*} & \sigma_{a,dLFI,dLWG}^{*} & \sigma_{a,dLFI,dBFTB}^{*} & \sigma_{a,dLFI,SMBW}^{*} \\ & \sigma_{a,dSWB}^{2*} & \sigma_{a,dSWB,dLWG}^{*} & \sigma_{a,dSWB,dBFTB}^{*} & \sigma_{a,dSWB,SMBW}^{*} \\ & & \sigma_{a,dLWG}^{2*} & \sigma_{a,dLWG,dBFTB}^{*} & \sigma_{a,dLWG,SMBW}^{*} \\ & & & \sigma_{a,dBFTB}^{2*} & \sigma_{a,dBFTB,SMBW}^{*} \\ & & & & \sigma_{a,SMBW}^{2*} \end{matrix} \right)$

Where,

$$\sigma_{a,dLFI}^{2*}= \sigma_{a,dLFI}^{2}$$

$$\sigma_{a,dLFI,dSWB}^{*}= \sigma_{a,dLFI}^{2}\times\lambda_{dSWB\leftarrow dLFI}{+ \sigma}_{a,dLFI,dSWB}+\sigma_{a,dLFI,dLWG}\times\lambda_{dSWB\leftarrow dLWG}$$

$$\sigma_{a,dLFI,dLWG}^{*}= \sigma_{a,dLFI,dLWG}$$

$$\sigma_{a,dLFI,dBFTB}^{*}= \sigma_{a,dLFI}^{2}\times\lambda_{dBFTB\leftarrow dSWB}\times\lambda_{dSWB\leftarrow dLFI}+\sigma_{a,dLFI,dSWB} \times\lambda_{dBFTB\leftarrow dSWB}+\sigma_{a,dLFI,dLWG}\times\lambda_{dBFTB\leftarrow dSWB}\times\lambda_{dSWB\leftarrow dLWG}+\sigma_{a,dLFI,dBFTB}$$

$$\sigma_{a,dLFI,SMBW}^{*}= \sigma_{a,dLFI}^{2}\times\lambda_{SMBW\leftarrow dSWB}\times\lambda_{dSWB\leftarrow dLFI}+\sigma_{a,dLFI,dSWB}\times\lambda_{SMBW\leftarrow dSWB}+ \sigma_{a,dLFI,dLWG}\times\lambda_{SMBW\leftarrow dSWB}\times\lambda_{dSWB\leftarrow dLWG}+\sigma_{a,dLFI,SMBW}$$

$$\sigma_{a,dSWB}^{2*}=\left( \lambda_{dSWB\leftarrow dLFI}\times\sigma_{a,dLFI}^{2}+\sigma_{a,dLFI,dSWB}+\lambda_{dSWB\leftarrow dLWG}\times\sigma_{a,dLFI,dLWG} \right)\times\lambda_{dSWB\leftarrow dLFI}+ \lambda_{dSWB\leftarrow dLFI}\times\sigma_{a,dLFI,dSWB}+\sigma_{a,dSWB}^{2}+\lambda_{dSWB\leftarrow dLWG}\times\sigma_{a,dSWB,dLWG}+\left( \lambda_{dSWB\leftarrow dLFI}\times\sigma_{a,dLFI,dLWG}+\sigma_{a,dSWB,dLWG}+\lambda_{dSWB\leftarrow dLWG}\times\sigma_{a,dLWG}^{2} \right) \times\lambda_{dSWB\leftarrow dLWG}$$

$$\sigma_{a,dSWB,dLWG}^{*}= \sigma_{a,dLFI,dLWG}\times\lambda_{dSWB\leftarrow dLFI}+\sigma_{a,dLWG,dSWB}+\sigma_{a,dLWG}^{2} \times\lambda_{dSWB\leftarrow dLWG}$$

$$\sigma_{a,dSWB,dBFTB}^{*}=\left( \sigma_{a,dLFI}^{2}{\times\lambda}_{dSWB\leftarrow dLFI}+\sigma_{a,dLFI,dSWB}+ \sigma_{a,dLFI,dLWG}\times\lambda_{dSWB\leftarrow dLWG} \right) \times\lambda_{dBFTB\leftarrow dSWB}\times\lambda_{dSWB\leftarrow dLFI}+\left( \sigma_{a,dLFI,dSWB}{\times\lambda}_{dSWB\leftarrow dLFI}+\sigma_{a,dSWB}^{2}+\sigma_{a,dSWB,dLWG}{\times\lambda}_{dSWB\leftarrow dLWG} \right) \times\lambda_{dBFTB\leftarrow dSWB}+\left( \sigma_{a,dLFI,dLWG}{\times\lambda}_{dSWB\leftarrow dLFI}+\sigma_{a,dSWB,dLWG}+ \sigma_{a,dLWG}^{2}{\times\lambda}_{dSWB\leftarrow dLWG} \right) \times\lambda_{dBFTB\leftarrow dSWB}\times\lambda_{dSWB\leftarrow dLWG}+ \sigma_{a,dLFI,dBFTB}{\times\lambda}_{dSWB\leftarrow dLFI}+\sigma_{a,dSWB,dBFTB}+ \sigma_{a,dLWG,dBFTB}{\times\lambda}_{dSWB\leftarrow dLWG}$$

$$\sigma_{a,dSWB,SMBW}^{*}=\left( \sigma_{a,dLFI}^{2} \times\lambda_{dSWB\leftarrow dLFI}+\sigma_{a,dLFI,dSWB}+\sigma_{a,dLFI,dLWG} \times\lambda_{dSWB\leftarrow dLWG} \right){\times\lambda}_{SMBW\leftarrow dSWB} \times\lambda_{dSWB\leftarrow dLFI}+ \left( \sigma_{a,dLFI,dSWB} \times\lambda_{dSWB\leftarrow dLFI}+\sigma_{a,dSWB}^{2}+\sigma_{a,dSWB,dLWG} \times\lambda_{dSWB\leftarrow dLWG} \right) \times\lambda_{SMBW\leftarrow dSWB}+ \left( \sigma_{a,dLFI,dLWG}\times\lambda_{dSWB\leftarrow dLFI}+\sigma_{a,dSWB,dLWG}+\sigma_{a,dLWG}^{2}\times\lambda_{dSWB\leftarrow dLWG} \right) \times\lambda_{SMBW\leftarrow dSWB}\times\lambda_{dSWB\leftarrow dLWG}+\sigma_{a,dLFI,SMBW}\times\lambda_{dSWB\leftarrow dLFI}+\sigma_{a,dSWB,SMBW}+\lambda_{dSWB\leftarrow dLWG}\times\sigma_{a,dLWG,SMBW}$$

$$\sigma_{a,dLWG}^{2*}= \sigma_{a,dLWG}^{2}$$

$$\sigma_{a,dLWG,dBFTB}^{*}= \sigma_{a,dLFI,dLWG}\times\lambda_{dBFTB\leftarrow dSWB}\times\lambda_{dSWB\leftarrow dLFI}+\sigma_{a,dSWB,dLWG}\times\lambda_{dBFTB\leftarrow dSWB}+\sigma_{a,dLWG}^{2}\times\lambda_{dBFTB\leftarrow dSWB} \times\lambda_{dSWB\leftarrow dLWG}+\sigma_{a,dLWG,dBFTB}$$

$$\sigma_{a,dLWG,SMBW}^{*}= \sigma_{a,dLFI,dLWG}\times\lambda_{SMBW\leftarrow dSWB}\times\lambda_{dSWB\leftarrow dLFI}+\sigma_{a,dSWB,dLWG}\times\lambda_{SMBW\leftarrow dSWB}+\sigma_{a,dLWG}^{2}\times\lambda_{SMBW\leftarrow dSWB}\times\lambda_{dSWB\leftarrow dLWG}+\sigma_{a,dLWG,SMBW}$$

$$\sigma_{a,dBFTB}^{2*}=\left( \lambda_{dBFTB\leftarrow dSWB}\times\lambda_{dSWB\leftarrow dLFI} \times\sigma_{a,dLFI}^{2}+\lambda_{dBFTB\leftarrow dSWB}\times\sigma_{a,dLFI,dSWB}+\lambda_{dBFTB\leftarrow dSWB} \times\lambda_{dSWB\leftarrow dLWG} \times\sigma_{a,dLFI,dLWG}+\sigma_{a,dLFI,dBFTB} \right)\times\lambda_{dBFTB\leftarrow dSWB} \times\lambda_{dSWB\leftarrow dLFI}+\left( \lambda_{dBFTB\leftarrow dSWB} \times\lambda_{dSWB\leftarrow dLFI} \times\sigma_{a,dLFI,dSWB}+ \lambda_{dBFTB\leftarrow dSWB}\times\sigma_{a,dSWB}^{2}+\lambda_{dBFTB\leftarrow dSWB}\times\lambda_{dSWB\leftarrow dLWG} \times\sigma_{a,dSWB,dLWG}+\sigma_{a,dSWB,dBFTB} \right) \times\lambda_{dBFTB\leftarrow dSWB}+\left( \lambda_{dBFTB\leftarrow dSWB}\times\lambda_{dSWB\leftarrow dLFI}\times\sigma_{a,dLFI,dLWG}+\lambda_{dBFTB\leftarrow dSWB}\times\sigma_{a,dSWB,dLWG}+\lambda_{dBFTB\leftarrow dSWB}\times\lambda_{dSWB\leftarrow dLWG}\times\sigma_{a,dLWG}^{2}+\sigma_{a,dLWG,dBFTB} \right)\times\lambda_{dBFTB\leftarrow dSWB} \times\lambda_{dSWB\leftarrow dLWG}+\lambda_{dBFTB\leftarrow dSWB}\times\lambda_{dSWB\leftarrow dLFI}\times\sigma_{a,dLFI,dBFTB}+\lambda_{dBFTB\leftarrow dSWB} \times\sigma_{a,dSWB,dBFTB}+\lambda_{dBFTB\leftarrow dSWB} \times\lambda_{dSWB\leftarrow dLWG}\times\sigma_{a,dLWG,dBFTB}+\sigma_{a,dBFTB}^{2}$$

$$\sigma_{a,dBFTB,SMBW}^{*}= \left( \lambda_{dBFTB\leftarrow dSWB}\times\lambda_{dSWB\leftarrow dLFI}\times\sigma_{a,dLFI}^{2}+\lambda_{dBFTB\leftarrow dSWB}\times\sigma_{a,dLFI,dSWB}+\lambda_{dBFTB\leftarrow dSWB}\times\lambda_{dSWB\leftarrow dLWG}\times\sigma_{a,dLFI,dLWG}+\sigma_{a,dLFI,dBFTB} \right)\times\lambda_{SMBWB\leftarrow dSWB} \times\lambda_{dSWB\leftarrow dLFI}+\left( \lambda_{dBFTB\leftarrow dSWB}\times\lambda_{dSWB\leftarrow dLFI}\times\sigma_{a,dLFI,dSWB}+ \lambda_{dBFTB\leftarrow dSWB}\times\sigma_{a,dSWB}^{2}+\lambda_{dBFTB\leftarrow dSWB} \times\lambda_{dSWB\leftarrow dLWG}\times\sigma_{a,dSWB,dLWG}+\sigma_{a,dSWB,dBFTB} \right) \times\lambda_{SMBWB\leftarrow dSWB}+\left( \lambda_{dBFTB\leftarrow dSWB}\times\lambda_{dSWB\leftarrow dLFI}\times\sigma_{a,dLFI,dLWG}+\lambda_{dBFTB\leftarrow dSWB} \times\sigma_{a,dSWB,dLWG}+\lambda_{dBFTB\leftarrow dSWB}\times\lambda_{dSWB\leftarrow dLWG}\times\sigma_{a,dLWG}^{2}+\sigma_{a,dLWG,dBFTB} \right)\times\lambda_{SMBWB\leftarrow dSWB}\times\lambda_{dSWB\leftarrow dLWG}+\lambda_{dBFTB\leftarrow dSWB}\times\lambda_{dSWB\leftarrow dLFI}\times\sigma_{a,dLFI,dBFTB}+\lambda_{dBFTB\leftarrow dSWB} \times\sigma_{a,dSWB,SMBW}+\lambda_{dBFTB\leftarrow dSWB}\times\lambda_{dSWB\leftarrow dLWG}\times\sigma_{a,dLWG,SMBW}+\sigma_{a,dBFTB,SMBW}$$

$$\sigma_{a,SMBW}^{2*}= \left( \lambda_{SMBWB\leftarrow dSWB}\times\lambda_{dSWB\leftarrow dLFI}\times\sigma_{a,dLFI}^{2}+\lambda_{SMBWB\leftarrow dSWB}\times\sigma_{a,dLFI,dSWB}+\lambda_{SMBWB\leftarrow dSWB}\times\lambda_{dSWB\leftarrow dLWG}\times\sigma_{a,dLFI,dLWG}+\sigma_{a,dLFI,SMBW} \right)\times\lambda_{SMBWB\leftarrow dSWB} \times\lambda_{dSWB\leftarrow dLFI}+\left( \lambda_{SMBWB\leftarrow dSWB} \times\lambda_{dSWB\leftarrow dLFI}\times\sigma_{a,dLFI,dSWB}+\lambda_{SMBWB\leftarrow dSWB}\times\sigma_{a,dSWB}^{2}+\lambda_{SMBWB\leftarrow dSWB}\times\lambda_{dSWB\leftarrow dLWG}\times\sigma_{a,dSWB,dLWG}+\sigma_{a,dSWB,SMBW} \right) \times\lambda_{SMBWB\leftarrow dSWB}+\left( \lambda_{SMBWB\leftarrow dSWB}\times\lambda_{dSWB\leftarrow dLFI}\times\sigma_{a,dLFI,dLWG}+\lambda_{SMBWB\leftarrow dSWB}\times\sigma_{a,dSWB,dLWG}+\lambda_{SMBWB\leftarrow dSWB} \times\lambda_{dSWB\leftarrow dLWG}\times\sigma_{a,dLWG}^{2}+\sigma_{a,dLWG,SMBW} \right)\times\lambda_{SMBWB\leftarrow dSWB} \times\lambda_{dSWB\leftarrow dLWG}+\lambda_{SMBWB\leftarrow dSWB}\times\lambda_{dSWB\leftarrow dLFI}\times\sigma_{a,dLFI,dBFTB}+\lambda_{SMBWB\leftarrow dSWB} \times\sigma_{a,dSWB,SMBW}+\lambda_{SMBWB\leftarrow dSWB}\times\lambda_{dSWB\leftarrow dLWG}\times\sigma_{a,dLWG,SMBW}+\sigma_{a,SMBWB}^{2}$$
